# CD13 promotes hepatocellular carcinogenesis and sorafenib resistance by activating HDAC5‐LSD1‐NF‐κB oncogenic signaling

**DOI:** 10.1002/ctm2.233

**Published:** 2020-12-01

**Authors:** Bo Hu, Yang Xu, Yuan‐Cheng Li, Jun‐Feng Huang, Jian‐Wen Cheng, Wei Guo, Yue Yin, Yang Gao, Peng‐Xiang Wang, Sui‐Yi Wu, Jian Zhou, Jia Fan, Xin‐Rong Yang

**Affiliations:** ^1^ Department of Liver Surgery & Transplantation Liver Cancer Institute Zhongshan Hospital, Fudan University Shanghai P.R. China; ^2^ Key Laboratory of Carcinogenesis and Cancer Invasion Ministry of Education Shanghai P. R. China; ^3^ Institutes of Biomedical Sciences Fudan University Shanghai P.R. China; ^4^ Department of Intensive Care Medicine Zhongshan Hospital Fudan University Shanghai P. R. China; ^5^ Department of Laboratory Medicine Zhongshan Hospital Fudan University Shanghai P. R. China

**Keywords:** CD13, HDAC5, hepatocellular carcinoma, patient derived tumor grafts, sorafenib‐resistance

## Abstract

**Rationale:**

CD13 is a new marker for liver cancer stem cells (CSCs) that contributes to sorafenib resistance in hepatocellular carcinoma (HCC). However, the underlying mechanism of CD13 in HCC sorafenib resistance remains enigmatic.

**Methods:**

The expression of CD13 in HCC cell lines and tissues was assayed by RT‐PCR, western‐blot, and immunohistochemistry staining. Athymic BALB/c nu/nu mice model was used to study the in vivo functions of CD13. Clinical significance of CD13 was evaluated by Kaplan‐Meier methods. Cellular proliferation rate was evaluated by cell counting kit‐8 cell proliferation assay and colony formation assay. Tunel assay was used to detect cell death ratio. Transwell assay was used to evaluate the motility of cells. Immunoprecipitation (IP), liquid chromatography‐mass spectrometry (LC‐MS)/MS, and co‐IP were applied to investigate potential protein interactions of CD13.

**Results:**

In this research, we found that CD13 expression was higher in metastatic HCC samples, and its overexpression was predicted worse prognosis for patients after surgical resection. Functionally, CD13 promoted HCC proliferation, invasion, cell cycle progression as well as sorafenib resistance. Mechanistically, CD13 interacted with histone deacetylase5 (HDAC5) to promote its protein stability, thus resulting in HDAC5‐mediated lysine‐specific demethylase 1 (LSD1) deacetylation and protein stabilization. Consequently, LSD1 decreased the NF‐κB catalytic unit p65 methylation that led to p65 protein stability. A CD13 inhibitor ubenimex in combination with sorafenib, suppressed the tumor growth and attenuated the resistance of HCC cells toward sorafenib in patient‐derived xenograft models.

**Conclusions:**

CD13 promotes HCC progression and induces sorafenib resistance, mainly via interacting with HDAC5 to prevent the degradation of p65 and activate NF‐kB signaling pathway. CD13 is a prognostic indicator for HCC patients underwent curative resection as well as a predictor of response to treatment with sorafenib. Our study establishes the new therapeutic potential of targeting CD13‐HDAC5‐LSD1‐NF‐κB in HCC.

## Graphical abstract

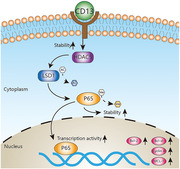

**Abbreviations**
AFP: alpha‐fetoprotein
CHX: cycloheximide
CNLC: China liver cancer staging
Co‐IP: co‐immunoprecipitation
CSCs: cancer stem cells
FCM: flow cytometry
HCC: hepatocellular carcinoma
HDAC5: histone deacetylase5
IHC: immunohistochemistry
IP: Immunoprecipitation
IRS: immunoreactive score system
KM: Kaplan‐Meier
LC‐MS: liquid chromatography–mass spectrometry
LSD1: lysine‐specific demethylase1
OS: overall survival
PDX: patient‐derived xenograft
SR: sorafenib‐resistant
TKI: tyrosine kinase inhibitor
TMAs: tissue microarrays
TTR: time‐to‐relapse
WB: western blot

## 1 BACKGROUND

Hepatocellular carcinoma (HCC) represents about 75‐85% of primary liver cancer, which ranks the fourth leading cause of cancer death worldwide.^1^ Surgery is still the most effective and potential treatment for liver cancer, while less than 30% of HCC patients are eligible for surgical intervention.^2^ Despite that multi‐tyrosine kinase inhibitor (TKI) sorafenib was approved as the standard treatment for advanced HCC, the survival benefit is modest.^3^ Furthermore, patients with HCC undergoing TKI inevitably develop resistance within several months, leading to high incidences of disease progression and distant metastasis.^4^, ^5^, ^6^ Therefore, it is critical to understand the mechanisms contributing to sorafenib resistance in order to develop novel therapeutic strategies to treat HCCs.

Previous studies demonstrated that cancer stem cells (CSCs), a small subpopulation of cells bearing progenitor cell‐like features, possessed both self‐renewal and differentiation capabilities,^7^ therefore contributing to tumor relapse, metastasis, drug resistance, and eventual patient mortality.^8^ Recently studies reported the correlation between sorafenib resistance and tumor cell subpopulations with stem/progenitor cell phenotypes in HCC, implicating that targeting these subpopulations might be a promising option for overcoming drug resistance in HCC.^9^ Aminopeptidase N (also named CD13) is a widely expressed type II membrane‐bound metalloprotease involved in pleiotropic functions, such as cell adhesion, proliferation, and motility.^10^ Recent research demonstrated that CD13 is a novel marker for semi‐quiescent CSCs in HCC and is responsible for the chemoresistance.^11^ Although CD13 may be a potential therapeutic target for alleviating drug resistance in HCC therapy, the potential role and underlying molecular regulation mechanism by which CD13 contributes to the sorafenib resistance remain poorly understood.

In this research, we measured the effects of CD13 on tumor cell proliferation, cell cycle, and migration, invasion and xenograft tumor growth. We also examined the molecular mechanism by which CD13 contributes to these processes by using integrated approaches including gene expression profiling, co‐immunoprecipitation, and liquid chromatography‐mass spectrometry (LC‐MS/MS). The antitumor effect of ubenimex, a CD13 inhibitor used in the clinic, was measured in both sorafenib‐resistant (SR) and sensitive HCC cell lines as well as patient‐derived xenografts (PDX). Our study provides the molecular mechanism as well as the therapeutic potential of targeting CD13 in HCC, which opens new therapeutic avenues in this lethal cancer.

## 2 METHODS

### 2.1 Patients and samples

For PCR and western blot (WB) analysis, tumor tissue from 21 HCC patients with curative resection in January 2015 in our institute was collected. For evaluation of the clinical significance of CD13 in HCC cohort, 403 HCC patients who received curative resection from 2000 to 2002 in Zhongshan Hospital were recruited in present study. The diagnosis of HCC was based on histopathology. The inclusion and exclusion criteria were described as follows: (a) HCC diagnosis definition by pathology according to WHO criteria; (b) no previous anti‐cancer treatment; (c) surgical resection was defined as complete removal of all tumor nodules, and no tumor was found on the incision surface by histological examination; (d) availability of appropriate formalin fixation, paraffin embedded tissues, and frozen tissues; and (e) the availability of complete clinicopathologic and follow‐up data. The use of human subjects was approved by the research ethics committee of Zhongshan Hospital. Informed consent was obtained from all patients. The China liver cancer staging (CNLC) system^2^ was used to assess tumor stage.

Moreover, during March 2013 and October 2014, 45 advanced recurrent HCC patients received sorafenib treatment who had undergone liver resection prior to the treatment were recruited to evaluate the predictive value of CD13 for sorafenib resistance. These patients were monitored postsurgically until March 2017.

### 2.2 Drugs, HCC cell lines, and animal model

A stock solution of sorafenib was purchased from Bayer Pharmaceutical Corporation. CD13 inhibitor ubenimex (S1591) were purchased from Selleckchem. The human HCC cell lines MHCC97L, MHCC97H, HCCLM3 (Liver Cancer Institute, Fudan University, China), HepG2, PLC/PRF/5 (American Type Culture Collection), Bel‐7402, Huh‐7, and the non‐neoplastic cells L‐02 (Shanghai Institute of Cell Biology, Chinese Academy of Sciences) were maintained in DMEM (Dulbecco's Modified Eagle Medium, Gibco BRL, Grand Island, NY) with 10% heat‐inactivated fetal bovine serum (Gibco BRL, Grand Island, NY) at 37°C with 5% CO_2_. Establishment of PDXs and animal assay were approved by Zhongshan Hospital Research Ethical Committee. All the experiments in xenografts are described in Supporting Information Materials and Methods.

**Highlights**

- CD13 expression is higher in more metastatic HCC samples and its overexpression predicts worse prognosis in HCC patients.
- CD13 regulates HCC cell proliferation, invasion, primary tumor growth and sorafenib resistance.
- CD13‐HDAC5‐LSD1‐NF‐#x003BA;B signaling axis is a newly identified signaling axis in HCC.
- CD13 inhibitor Ubenimex restored sorafenib sensitivity in HCC.

### 2.3 CD13 positive cell sorting using immunomagnetic beads and flow cytometric analysis

After single‐cell suspension, 1 × 10^8^ HCC cells were incubated with CD13 immunomagnetic beads for 30 minutes at 4°C. According to manufacturer's instructions, CD13 positive (CD13^+^) cells linked with beads will be captured by MACS cell separation column. After centrifuge, CD13^+^ cells were resuspended in DMEM (with 10% fetal bovine serum).

CD13^+^ and CD13^−^ tumor cells, separated by MACS approach, were incubated with CD13 antibody on ice for 40 minutes in ice‐cold PBS with 1% FBS, and further validated by flow cytometry (FCM) according to manufacturer's instructions. Briefly, tumor cells were stained with PE‐conjugated CD13 monoclonal antibody (BD bioscience, San Jose, CA) for 30 minutes. FCM was done on a BD FACS Caliber. Analysis of FACS data was done using Flowjo software (v9, Tresstar). Isotype‐matched antibodies were used with all the samples as controls.

### 2.4 Real‐time PCR, western blot, and tunel assay

Real‐time PCR (Polymerase Chain Reaction) and immune blot were performed as previously described.^12^ The sequence of primers for PCR used is listed in Table S1. Antibodies used for WB assays were listed in Table S2. Cell proliferation and apoptosis were determined by Cyclin A and Cyclin D1 immunofluorescence staining and tunel assay (#11684795910, In Situ Cell Death Detection Kit Fluorescein, Roche), respectively, according to manufacturer's instructions.

### 2.5 Immunoprecipitation, LC‐MS/MS, and co‐immunoprecipitation

IP, LC‐MS/MS (Liquid Chromatography Mass Spectrometry), and co‐IP were described in the Supporting Materials and Methods.

### 2.6 Matrigel invasion assay, CCK‐8 assay, colony formation assay, and cell cycle assay

Matrigel invasion assay was performed to evaluate cell invasion activity, while CCK‐8 and colony formation assay were performed to evaluate cell proliferation activity. Cell cycle assay was performed as previously described.^13^ Details are shown in the Supporting Materials and Methods.

### 2.7 Construction of tissue microarrays and immunohistochemistry

Tissue microarrays (TMAs) were constructed as previously described.^14^ Antibody list is shown in Table S2. The immunoreactive score system was described previously.^15^ Details are shown in the Supporting Materials and Methods.

### 2.8 Cignal finder RTK signaling 10‐pathway reporter array

Cignal finder Receptor Tyrosine Kinase (RTK) signaling 10‐Pathway Reporter Array was used for identifying critical downstream signaling pathways of CD13 as previously described.^13^ Transfection of luciferase reporter plasmids and determination of altered signaling were performed according to manufacturer's instructions.

### 2.9 Cycloheximide protein half‐life assay

Note that 2 × 10^6^ HCC cells were treated with cycloheximide (CHX) (10 μg/mL) for 0, 0.5, 1, and 2 hours. Histone deacetylase5 (HDAC5) and p65 protein expressions were assessed by WB assays.

### 2.10 Ubiquitylation assay

Ubiquitylation assay was conducted according to previous study.^16^ Briefly, whole cell lysates were incubated with HDAC5, lysine‐specific demethylase (LSD1), or p65 antibodies individually at 4°C overnight, then incubate with Magnetic beads for 2 hours. WB examined the ubiquitin level of target protein with anti‐ubiquitin antibodies.

### 2.11 Statistical analysis

SPSS 20.0 for Mac was used for Statistical analyses. Quantitative data were presented as mean ± standard error (SE) of at least three independent experiments. Continuous data were analyzed by one‐way ANOVA and Student's t‐test, and categorical data were analyzed by Fisher's exact test or chi‐square test. Kaplan‐Meier (KM) method was used to calculate the cumulative recurrence rate and overall survival (OS) rate, and log rank test was used to evaluate the difference between the groups. Univariate and multivariate analyses were calculated by the Cox proportional hazards regression model. *P* < .05 was considered statistically significant.

## 3 RESULTS

### 3.1 CD13 expression level positively correlates with the metastatic potential and tumor recurrence in HCC

**FIGURE 1:**
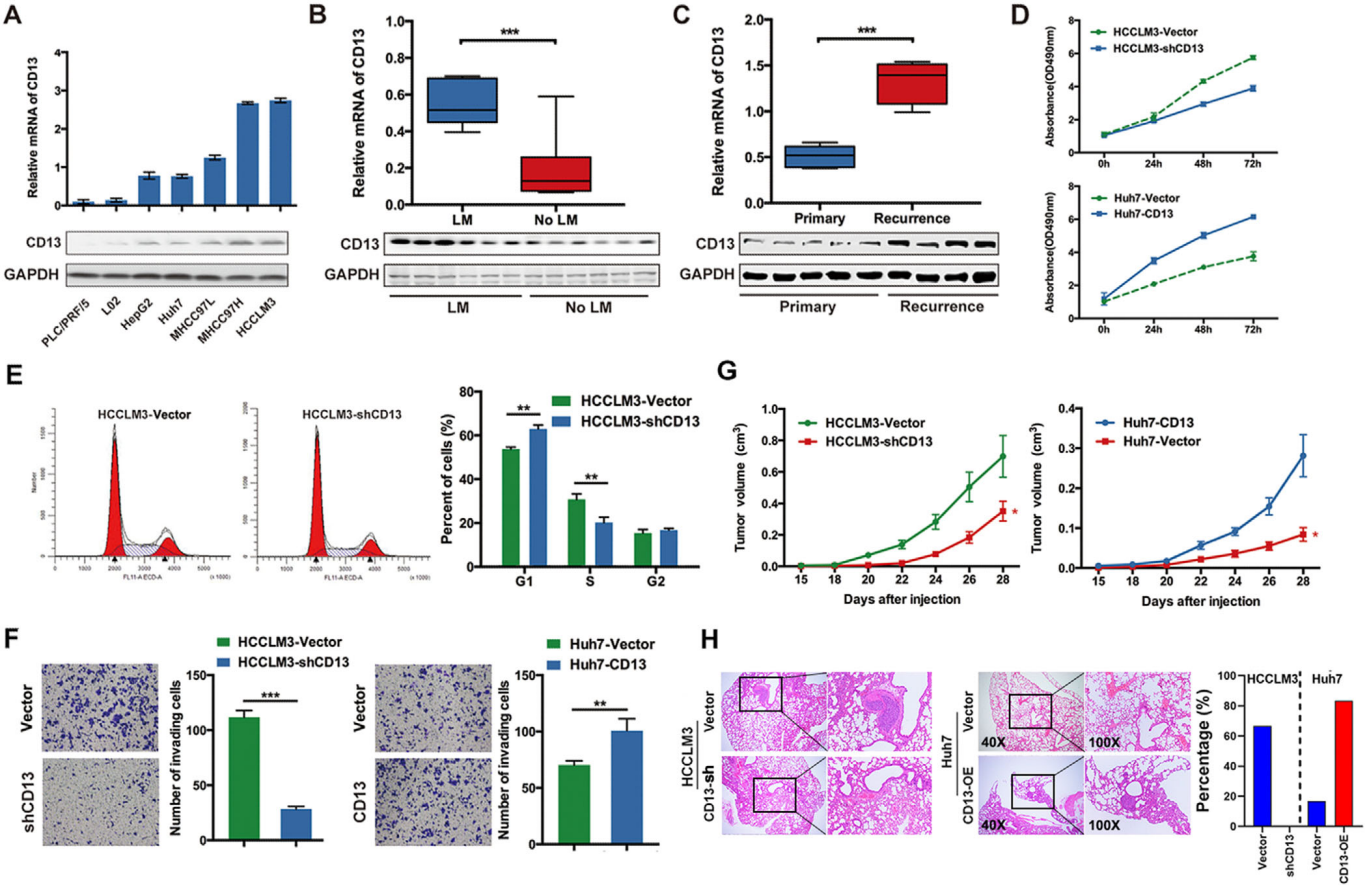
Biological functions of CD13 in HCC. A, mRNA (upper) and protein (lower) expressions of CD13 in HCC cell lines. L02, a normal hepatocyte cell line was used as a control. B, Protein level of CD13 in primary HCC tissues with/without lung metastasis. C, Protein level of CD13 in primary and recurrent HCC tissues. D, CD13 knock down significantly restrained HCCLM3 proliferation capacities, whereas CD13 overexpression greatly promoted Huh7 proliferation according to CCK‐8 assays. E, CD13 knock down resulted in G0/G1 arrest in HCCLM3 cells according to cell cycle determinations. F, CD13 knock down significantly inhibited HCCLM3 invasiveness capacities, whereas CD13 overexpression greatly enhanced Huh7 invasion according to transwell assays. G, CD13 knock down significantly hindered HCC xenografts growth (left), whereas CD13 overexpression greatly promoted HCC xenografts growth (right) according to subcutaneous tumor models. H, CD13 knock down reduced incidences of lung metastasis of HCC according to tail intravenous injection models, whereas CD13 overexpression greatly enhanced incidences of lung metastasis. *P < .05, **P < .01, ***P < .001

To examine the mRNA and protein levels of CD13 in HCC, firstly we performed qRT‐PCR and WB analyses in a panel of HCC cell lines, ranging from low metastatic potential (such as PLC/PRF/5, L02, HepG2, Huh7) to high metastatic potential (such as MHCC97L, MHCC97H, and HCCLM3). More metastatic cell lines displayed higher mRNA as well as protein levels of CD13 (Figure 1A). In accordance with these findings in cells, primary HCC tissues from patients suffering lung metastases showed higher levels of CD13 expression compared to those patients that did not have lung metastases (Figure 1B). Consistently, recurrent HCC tissues exhibited significantly higher expression of CD13 than primary HCC tissues (Figure 1C). These findings suggest the potential correlation between CD13 and HCC progression.

### 3.2 CD13 promotes oncogenic phenotypes in HCC

Motivated by the findings above, we aim to examine the role of CD13 in HCC. To this end, firstly we depleted CD13 in metastatic HCC cell lines HCCLM3 and MHCC97H by using the CD13 shRNA. To complement our loss‐of‐function studies, we also overexpressed CD13 in less‐metastatic cell lines such as Huh7 and HepG2. Modulations of CD13 expression were verified by RT‐PCR and WB assays (Figure S1A). Downregulation of CD13 expression suppressed tumor cell proliferation significantly in both HCCLM3 and MHCC97H cells, whereas upregulation of CD13 expression promoted cell proliferation in Huh7 and HepG2 cells (all *P* < .01; Figure 1D and Figure S1B). Consistent with this phenotype, cell cycle analysis showed increased G1 phase and correspondingly decreased S phase in cells with CD13 depletion (both *P* < .01, Figure 1E and Figure S1C). These results suggest that CD13 promotes HCC proliferation via promoting G1‐S cell cycle transition. In addition, we also examined the potential effect of CD13 in HCC invasion by Boyden chamber trans‐well assays and found that CD13 depletion led to decreased cell invasion in HCCLM3 and MHCC97H cells (both *P* < .001, Figure 1F and Figure S1D), whereas CD13 overexpression in Huh7‐ and HepG2 enhanced invasive potential, which is significantly compared with the control groups (both *P* < .01, Figure 1F and Figure S1D).

Next, we performed in vivo experiments to examine the role of CD13 in HCC xenografts. Consistent with our results obtained with cell lines in vitro, HCC cells with CD13 knockdown exhibited decreased tumor growth in vivo. In contrary, overexpression of CD13 significantly promoted tumor growth (all *P* < .01, Figure 1G and Figure S2). We also performed H&E (Hematoxylin‐Eosin) staining for the lung tissues upon necropsy and found that CD13 knockdown significantly reduced the incidence of lung metastases (66.7% vs 0.0%, *P* < .001, Figure 1H), while upregulation of CD13 in Huh7 increased the incidence of lung metastases (16.7% vs 83.3%, *P* = .021, Figure 1H). Taken together, our results suggest that CD13 is both necessary and sufficient to HCC cell proliferation, invasion, and metastasis in HCC.

### 3.3 CD13 expression predicts HCC patients survival with curative resection and sorafenib treatment

**FIGURE 2:**
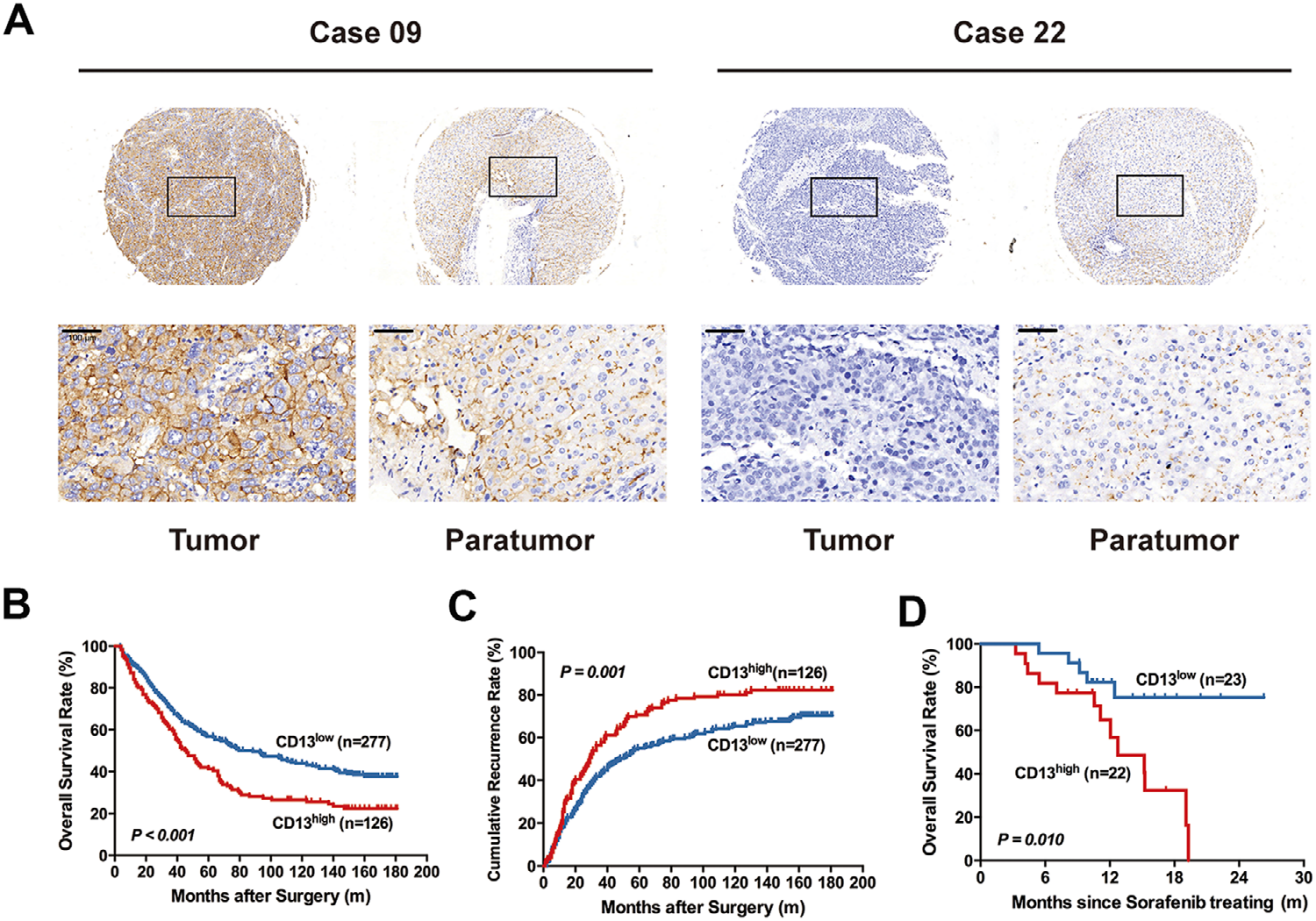
Clinical significance of CD13 in HCC patients. A, Representative images of CD13‐high (left) and CD13‐low (right) according to IHC staining. B, Kaplan‐Meier curve for overall survival in patients received curative resection according to their CD13 level. C, Kaplan‐Meier curve for time‐to‐recurrence in patients received curative resection according to their CD13 level. D, Kaplan‐Meier curve for overall survival in patients received sorafenib treatment according to their CD13 expression

The expression of CD13 was detected by IHC staining in TMAs containing 403 HCC patients. The main location of CD13 was on the plasma membrane of tumor cells and liver cells. The expression of CD13 in tumor tissues was classified as high (score > 4) in 126 cases (31.3%) and low (score 0‐4) in 277 cases (68.7%) (Figure 2A). We found that CD13^high^ patients displayed the clinical manifestations of elevated alpha‐fetoprotein (AFP), tumor encapsulation, and vascular invasion (All *P* value < .05; Table S3). Patient clinicopathologic characteristics are shown in Table S3.

As shown in KM analysis, HCC patients with high CD13 level were significantly correlated with higher recurrence as well as poor prognosis after operation (median time‐to‐relapse [TTR]: 29.0 vs 48.0 months, *P* < .001; median OS: 45.5 vs 88.5 months, *P* < 0.001; Figure 2B,C). Multivariate analyses demonstrated that high expression of CD13 was an independent predictor for OS (HR, 1.34; 95% CI, 1.03‐1.74; *P* = .029) and postoperative recurrence (HR, 1.33; 95% CI, 1.04‐1.71; *P* = .026; Table 1). Moreover, we found that the prognostic significance of CD13 was retained in AFP‐negative and early stage patients (CNLC stage I subgroups) (Figure S3). In the AFP ≤ 20 ng/mL group, the 5‐year TTR rate was 32.14% in CD13^+^ patients compared with 50.88% in CD13^−^ patients (*P* < .05), and 36.76% versus 52.88% in the CNLC stage I, respectively (*P* < .01).

**TABLE 1:** Univariate and multivariate Cox regression analyses of CD13 with clinic‐pathologic characteristics (n = 403)

Variables	OS				TTR	
	Univariate	Multivariate		Univariate	Multivariate	
	*P* value	HR (95% CI)	*P* value	*P* value	HR (95% CI)	*P* value
Age, years (>50 vs ≤50)	.858	NA	.787		NA	
Sex (male vs female)	.142	NA	.357		NA	
Liver cirrhosis (yes vs no)	.010	1.606 (1.126‐2.291)	.009	.001	1.644 (1.178‐2.296)	.003
Child‐Pugh (B vs A)	.353	NA	.249		NA	
HBsAg (positive vs negative)	.291	NA	.248		NA	
HCV (positive vs negative)	.991	NA	.857		NA	
GGT (>54 vs ≤54)	<.001	1.363 (1.043‐1.780)	.023	.001	1.333 (1.044‐1.702)	.021
ALT, U/l (>40 vs ≤40)	.825	NA	.935		NA	
AFP, ng/mL (>20 vs ≤20)	.007	1.223 (0.938‐1.596)	.137	.022	1.147 (0.891‐1.477)	.286
Tumor encapsulation (none vs complete)	.005	1.072 (0.823‐1.396)	.608	.029	1.010 (0.785‐1.301)	.936
Tumor differentiation (III‐IV vs I‐II)	.305	NA	.394		NA	
Tumor size, cm (>5 vs ≤5)	.010	1.171 (0.902‐1.519)	.236	.145	NA	
Tumor number (multiple vs single)	<.001	1.324 (0.974‐1.626)	.114	<.001	1.276 (0.942‐1.523)	.173
Vascular invasion (yes vs no)	<.001	1.125 (0.682‐1.856)	.643	<.001	1.274 (0.781‐2.080)	.332
CNLC (II‐III vs I)	<.001	2.865 (1.528‐5.375)	.001	<.001	3.054 (1.698‐5.494)	<.001
CD13 (high vs low)	<.001	1.337 (1.028‐1.740)	.029	.001	1.332 (1.035‐1.714)	**.026**

Abbreviations: AFP, alpha‐fetoprotein; ALT, alanine aminotransferase; CNLC, China liver cancer staging; GGT, gamma‐glutamyl transferase; HBsAg, hepatitis B surface antigen; HCV, hepatitis C virus; NA, not applicable.

The predictive value of CD13 expression for sorafenib resistance was further explored in 45 HCC patients received sorafenib treatment after tumor recurrence. Clinicopathologic characteristics are shown in Table S4. KM analysis showed that CD13^high^ patients had a significantly shorter OS compared with CD13^low^ patients after sorafenib treatment. Median OS was 12.7 months in the CD13^high^ group versus not reached in the CD13^low^ group (*P* = .010, Figure 2D).

### 3.4 CD13 induces sorafenib resistance and its blockade restores the sensitivity of sorafenib in HCC

**FIGURE 3:**
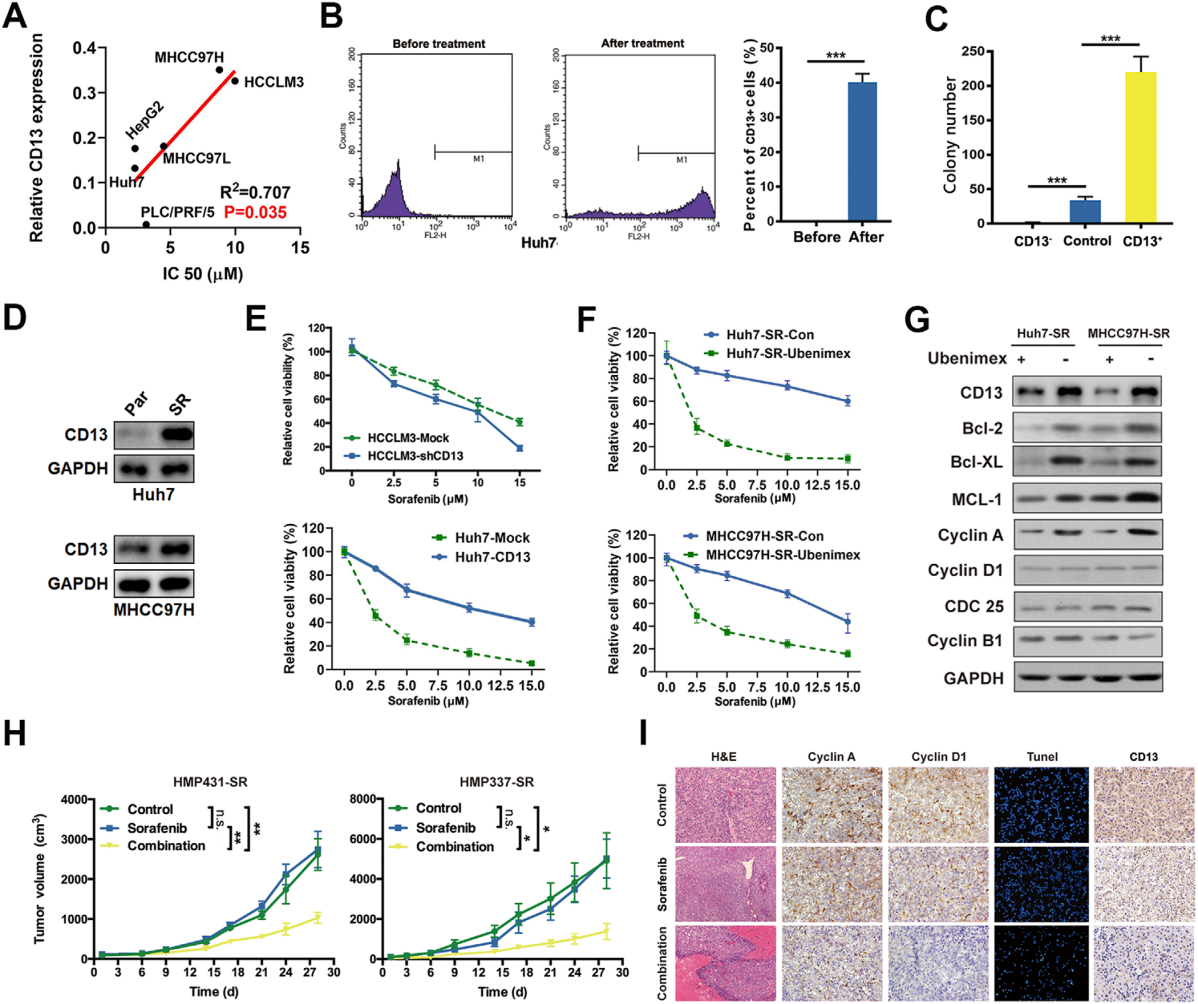
CD13 mediated sorafenib resistance and served as a promising target for re‐sensitization. A, Correlations between CD13 protein expression levels and IC50 for sorafenib in HCC cell lines. B, Fraction of CD13+ Huh7 cells before (left) and after (right) sorafenib treatment. C, CD13+ Huh7 cells exhibited greater proliferation potentials under sorafenib treatment than their CD73‐ counterparts and parental cells did. D, Protein expression of CD13 in sorafenib‐resistant and corresponding parental HCC cells. E, CD13 knockdown greatly sensitized HCCLM3 cells to sorafenib treatment, whereas forced CD13 expression induced sorafenib resistance in Huh7 cells according CCK‐8 assays. F, Effects of ubenimex treatment on drug re‐sensitization in sorafenib‐resistant cells were evaluated by CCK‐8 assays. G, Effects of ubenimex (0.25 mg/mL) treatment on anti‐apoptotic and cell cycle gene expression states were evaluated by WB assays. H, Evaluations of inhibitory efficiencies of sorafenib (30 mg/kg), or ubenimex (4 mg/kg), and sorafenib combination were conducted in two sorafenib‐resistant PDX models. I, HE, cyclin A, cyclin D1, tunel, and CD13 staining of the xenograft tumors (HMP337‐SR) are shown

Since the correlation of CD13 and sorafenib resistance was observed in clinical patients, we found that patients with high CD13 level displayed shorter survival with sorafenib treatment compared to those patients expressing low CD13. We found that the increased expression level of CD13 corresponded to elevated IC50 with sorafenib treatment in HCC cell lines (*R^2^* ^^= 0.707, *P* = .035, Figure 3A). Furthermore, the percentage of CD13^+^ cells in Huh7 was significantly increased after sorafenib treatment for 72 hours, suggesting the enrichment of CD13 subpopulation by sorafenib administration (Figure 3B). Meanwhile, we found that CD13^+^ tumor cells isolated from Huh7 after sorafenib treatment showed enhanced spheroid‐forming ability compared with CD13^−^ cells or parental Huh7 cells (Figure 3C). Furthermore, we examined CD13 mRNA and protein levels in the previously established SR Huh7 and MHCC97H cell lines,^17^, ^18^ and higher CD13 expression levels were observed in HCC‐SR cells compared with their correspondingly parental cells (*P* < .001, Figure 3D and Figure S4A).

Next, we examined the role of CD13 in sorafenib treatment responsiveness by loss‐of‐function with shRNAs and gain‐of‐function with overexpression experiments. Downregulation of CD13 expression in HCCLM3 and MHCC97H cells significantly enhanced their sensitivity to sorafenib treatment, whereas up‐regulation of CD13 in Huh7 and HepG2 cells significantly increased their drug resistance to sorafenib (*P *< .01, Figure 3E).

The chemical agent ubenimex, which is known as a CD13 inhibitor,^19^ was further used to explore the role of CD13 in sorafenib resistance. We found that ubenimex administration could restore the sensitivity of sorafenib on HCC cells to the similar extent as CD13 knockdown did (Figure 3F). Meanwhile, we found that the expressions of the critical cell cycle regulator such as cyclin A were significantly repressed by ubenimex administration in SR tumor cells (Figure 3G and Figure S4C). In addition, the levels of several anti‐apoptotic proteins such as BCL‐2 and MCL‐1 were also reduced by ubenimex (Figure 3G and Figure S4D).

Next, we also constructed two sorafenib‐acquired‐resistant PDX models (HMP431‐SR and HMP337‐SR), where CD13 expressed in both PDXs, and tested the effects of ubenimex on these two models in vivo. We found that untreated control or sorafenib‐treated PDXs showed the increased tumor growth overtime, whereas the tumor growth was significantly diminished in the mice with sorafenib treatment alone or in combination with ubenimex compared with the control groups in these two PDX models (Figure 3H and Figure S4B). On the other hand, no significant side effects, such as body weight loss, anorexia, and diarrhea, were observed in any ubenimex treatment groups (data not shown). Hematoxylin and eosin showed that necrosis was induced in cancer tissues by combination treatment. IHC staining for these tumors upon necropsy showed that Cyclin A, Cyclin D1, and CD13 were significantly decreased in combination group compared with either control or sorafenib group only. On the other hand, increased apoptosis by tunel staining was observed in the combination group (Figure 3I). Together, our results suggest that CD13 promotes sorafenib resistance.

### 3.5 CD13 promoted sorafenib resistance via activating NF‐κB signaling

**FIGURE 4:**
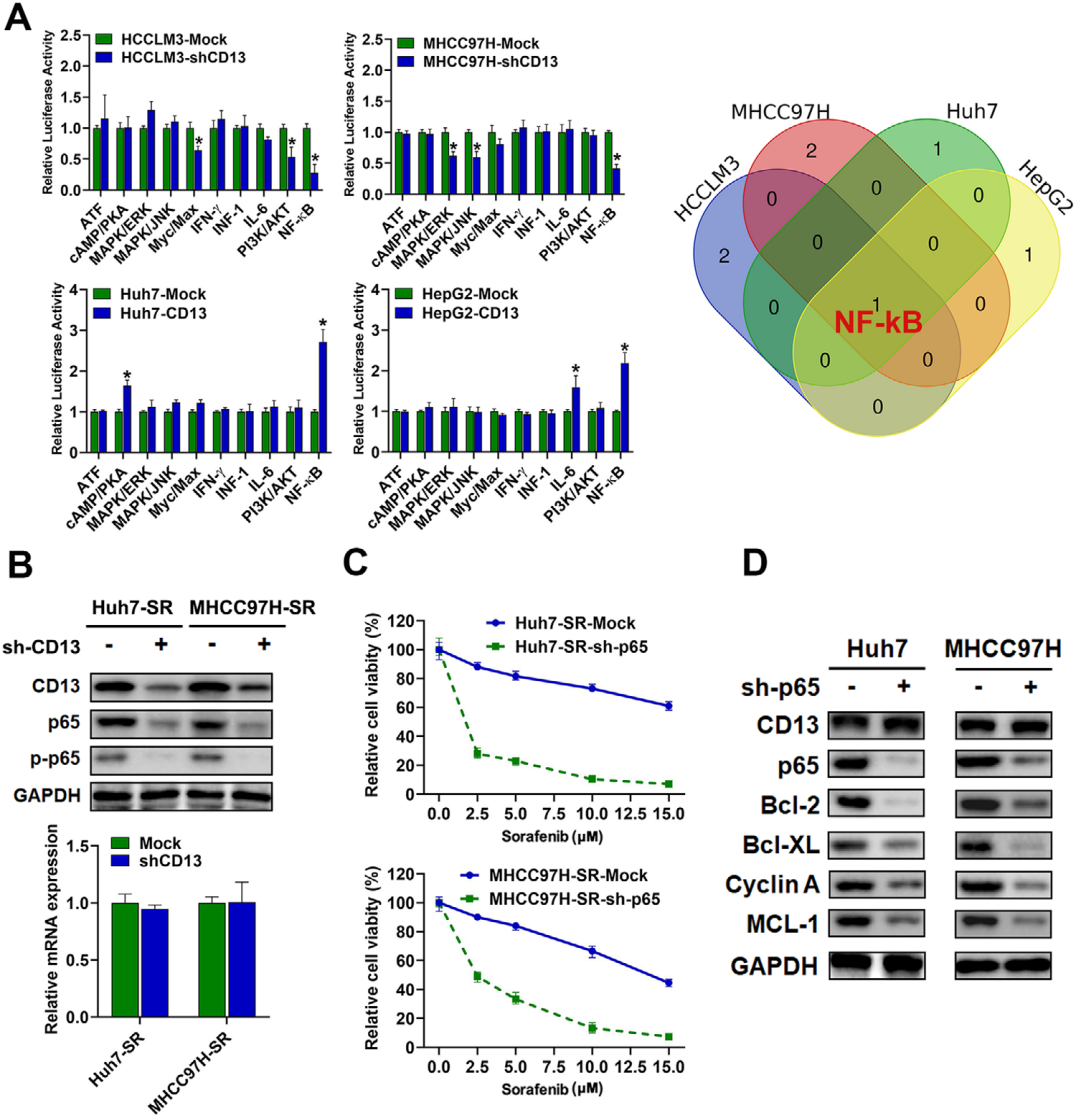
CD13 conferred sorafenib resistance to HCC via activating NF‐kB signaling. A, Critical downstream signaling of CD13 was screened via Cignal Finder Reporter Array, and NF‐kB signaling was identified as the critical downstream pathway responsible for CD13‐mediated sorafenib resistance in HCC. B, Protein and mRNA expressions of p65 and p‐p65 were detected by WB and RT‐PCR, respectively. C, p65 knockdown restored sorafenib susceptibility in HCC cells according to CCK‐8 assays. D, Effects of p65 knockdown on anti‐apoptotic and cell cycle gene expression states in sorafenib‐resistant HCC cells were assessed by western blot

Next, we aimed to determine the molecular mechanism by which CD13 contributes to HCC tumor progression as well as sorafenib resistance. Firstly, to discover the down‐stream signaling pathway regulated by CD13, we implemented Cignal Finder RTK signaling 10‐pathway reporter array. We found that NF‐κB signal pathway was the most consistently regulated pathway by CD13 modulation (Figure 4A), CD13 depletion in multiple HCC cell lines leading to decreased NF‐κB activity while its overexpression in these cell lines causing increased NF‐κB reporter activity. As the key protein involved in NF‐κB pathway,^20^, ^21^ the expression level of p65 and phosphorylated p65 (p‐p65) was detected by WB assays. We found that downregulated CD13 decreased p65 protein levels as well as phosphorylated p65 (p‐p65) significantly in Huh7‐SR and MHCC97H‐SR cells (Figure 4B) while not affecting p65 mRNA level, implying that post‐transcription modification may contribute to CD13‐mediated NF‐kB pathway activation. Similar results were observed in MHCC97H and HCCLM3 cells after CD13 knockdown (Figure S5A).

To examine the role of p65 on mediating the effect of CD13 and sorafenib resistance in Huh7‐SR and MHCC97H‐SR cells, we used the p65 shRNA to deplete its expression in these cells and found that p65 knockdown could resensitize the SR phenotype in these two HCC cell lines (Figure 4C). Consistently, p65 silencing also resulted in re‐sensitization of sorafenib in MHCC97H and HCCLM3 cells (Figure S5B). WB assays also confirmed that silencing p65 resulted in decreased protein levels of BCL‐2 (B‐cell lymphoma 2), Cyclin A, MCL‐1 (Myeloid cell leukemia 1), and Bcl‐XL (Figure 4D). Concordantly, downregulation of p65 in MHCC97H and HCCLM3 cells caused reduction of BCL‐2, Cyclin A, MCL‐1, and Bcl‐XL protein levels (Figure S5C).

### 3.6 HDAC5 is a new CD13 interactor to regulate NF‐kB activation and sustain sorafenib resistance in HCC

**FIGURE 5:**
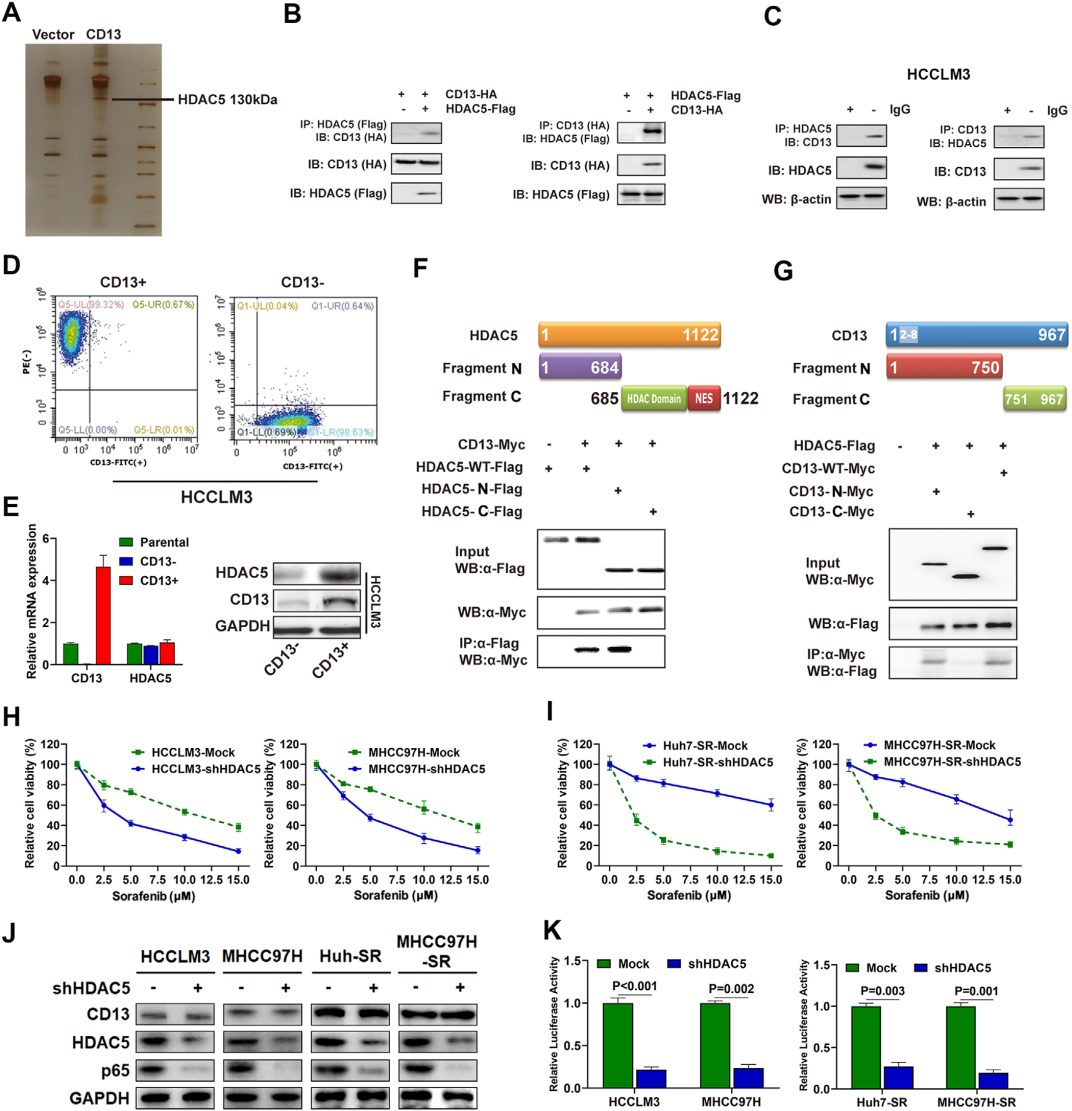
HDAC5 was identified as an essential interactor for CD13 to maintain CD13‐mediated sorafenib resistance. A, Silver staining of purified CD13 complex followed by mass spectrometry analysis. HDAC5 was identified to be a CD13‐interacting protein as indicated. B, Immunoprecipitation (IP) of 293T cells transfected with CD13‐HA and HDAC5‐Flag plasmids as indicated. C, IP of HCCLM3 cells for the endogenous interaction between HDAC5 and CD13. D, FACS sorting of HCCLM3 cells according to CD13 marker expression. E, mRNA and protein expressions of CD13 and HDAC5 in CD13+, CD13‐, and parental HCC cells were assessed by RT‐PCR and western blot, respectively. F, Upper: A diagram shows series of flag tagged truncation mutants of HDAC5;Lower: Immunoblot (IB) assays of whole cell extracts and IP of 293T cells transfected with CD13‐Myc or HDAC5‐WT‐Flag or truncation mutants. G, Upper: A diagram shows series of Myc tagged truncation mutants of CD13; Lower: IB assays of whole cell extracts and immunoprecipitation (IP) of 293T cells transfected with HDAC5‐FLAG or CD13‐WT‐Myc or truncation mutants. H, HDAC5 knockdown restored sorafenib susceptibility in HCC cells according to CCK‐8 assays. I, HDAC5 knockdown restored sorafenib susceptibility in sorafenib‐resistant cells according to CCK‐8 assays. J, Effects of HDAC5 knockdown on p65 protein levels in ordinary and sorafenib‐resistant HCC cells according to WB assays. K. HDAC5 knockdown significantly inactivated NF‐kB signaling in ordinary and sorafenib‐resistant HCC cells according to dual‐luciferase assays

Our results suggest that CD13 regulates p65 post‐transcriptionally. However, the mechanism by which CD13 regulates sorafenib resistance via p65/NF‐κB remains unclear. To address this, we performed the LC‐MS in an attempt to identify the CD13 interaction proteins. We performed the protein purification with the specific antibody recognizing CD13 with cell lysates harvested from vector cells or cells overexpressing CD13 followed by mass spectrometry. A total of 180 proteins were identified by LC‐MS approach, most of which were related with nucleus transcription and modification (Table S5). However, we did not retrieve p65 polypeptides from the mass spectrometry despite that we could detect the interaction between CD13 and p65 by co‐IP assay (Figure S6), suggesting the likelihood that CD13 may regulate p65 protein level indirectly. From the mass spectrometry CD13 interaction list, HDAC5 (Histone Deacetylase 5) family proteins (HDAC5, HDAC1, and HDAC6) were listed as several of TOP10 CD13 interacting proteins, which garnered our attentions (Table S5), suggesting the importance of HDACs in CD13‐regulated signaling event in HCC. Among all HDACs identified, HDAC5 ranked the highest score (Table S5), suggesting the strong potential of interaction between CD13 and HDAC5 in HCC cells, and HDAC5 may be a novel interactor of CD13 (Figure 5A).

Motivated by our mass spectrometry data, we aimed to determine the interaction between HDAC5 and CD13 in HCC cells. Further co‐IP assays conducted on 293T cells that transiently co‐expressed Flag‐HDAC5 and/or HA‐CD13 proteins confirmed that HDAC5 could interact with CD13 (Figure 5B). In addition, endogenous binding between HDAC5 and CD13 could also be detected by reciprocal immunoprecipitation in HCCLM3 cells (Figure 5C). Intriguingly, we found that HDAC5 expression was higher in CD13^+^ compared to CD13^−^ HCC cells by FACS (Figure 5D) despite that mRNA level of HDAC5 was comparable between these two cell populations (Figure 5E), suggesting the enhanced HDAC5 expression may mainly due to post‐transcriptional modification process caused by CD13.

The interaction regions between HDAC5 and CD13 proteins were explored by co‐IP assays in 293T cells, which were co‐expressed full‐length Myc‐CD13 and HDAC5 or their truncation mutants. We found that N‐terminal region of HDAC5 (1‐684aa) alone could bind with CD13 similarly as the full length HDAC5, whereas the other domains failed to bind CD13 (Figure 5F).

Moreover, we made serial truncations of CD13 and performed co‐IP assays. Notably, HDAC5 specifically bound to the N‐terminus of CD13 containing the cytoplasmic domain (2‐8) (Figure 5G). Collectively, these data demonstrate that N‐terminal region of HDAC5 (1‐684aa) specifically bound to CD13 dependent on cytoplasmic domain (2‐8). The role of HDAC5 in CD13‐mediated sorafenib resistance was further investigated, and we found that HDAC5 knockdown could enhance HCC cells to be sensitive toward sorafenib in cell lines with high CD13 expression (Figure 5H). Notably, knocking down HDAC5 in Huh7‐SR and MHCC97H‐SR cells also exerted similar effect on reversing sorafenib resistance (Figure 5I). Accordingly, WB and dual‐luciferase assays revealed that HDAC5 knockdown greatly restrained p65 protein expression as well as NF‐kB signal activation (Figure 5J,K).

### 3.7 CD13 promotes HDAC5 protein stability, therefore preventing p65 degradation and resulting in NF‐kB activation

**FIGURE 6:**
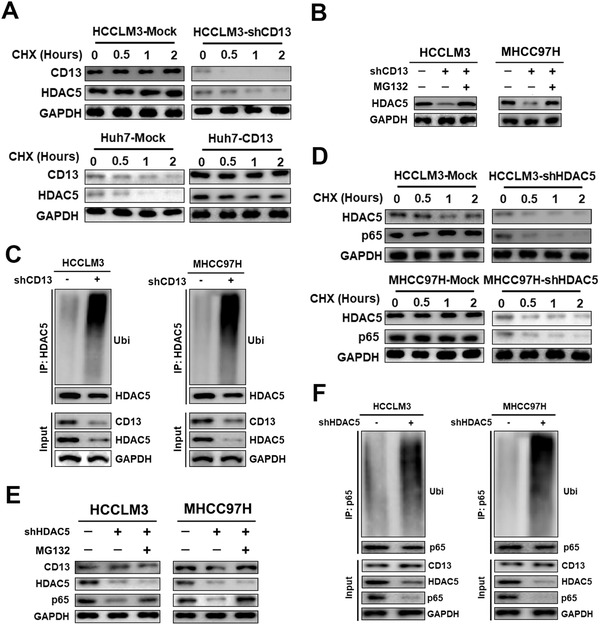
CD13 enhanced HDAC5 protein stability to upregulate p65 expression. A, Effects of CD13 knockdown (upper) and overexpression (lower) on the lifespan of HDAC5 protein were evaluated by CHX chasing assay. B, MG132 treatment could successfully restored HDAC5 expression in CD13 silenced HCC cells. C, Ubiquitination assay of HDAC5 in HCCLM3 (left) and MHCC97H (right) cells. Transfected cells were treated with MG132 for 6 hours. D, Effects of HDAC5 knockdown (upper) and overexpression (lower) on the lifespan of HDAC5 protein in CD13‐high HCC cells were evaluated by CHX chasing assay. E, MG132 treatment could successfully restored p65 expression in HDAC5 silenced CD13‐high HCC cells. F, Ubiquitination assay of p65 in HCCLM3 (left) and MHCC97H (right) cells after 6 hours treatment of MG‐132

Our results suggest that CD13 regulates HDAC5 post‐transcriptionally, we next examined whether HDAC5 protein stability can be regulated by CD13. To this end, we used CHX to inhibit the new protein synthesis and monitored HDAC5 protein stability over time in either HCCLM3‐mock cells or these cells depleted of CD13 by the shRNA. We found that the stability of HDAC5 was significantly decreased by CD13 knockdown (Figure 6A). Conversely, we also overexpressed CD13 in Huh7 cells and found that CD13 overexpression led to increased HDAC5 protein stability (Figure 6A). We also treated two different HCC cell lines depleted of CD13 with the proteasomal inhibitor MG132 and found that decreased HDAC5 protein levels in these cells were ameliorated by MG132, strengthening that CD13 regulates HDAC5 protein stability via preventing HDAC5 ubiquitination (Figure 6B). Consistent with this notion, CD13 knockdown in multiple HCC cells led to increased HDAC5 ubiquitination (Figure 6C).

Next, we aimed to validate whether HDAC5 could regulate p65 protein expression. Results showed that HDAC5 knockdown significantly decreased p65 protein stability in HCCLM3 and MHCC97H cell lines (Figure 6D), whereas MG132 treatment effectively rescued downregulation of p65 protein expression caused by HDAC5 silence (Figure 6E). Moreover, we observed greater ubiquitination level of p65 caused by HDAC5 knockdown in HCCLM3 and MHCC97H cell lines (Figure 6F). In summary, our findings suggest that CD13 promotes HDAC5 and p65 protein stability, therefore activating NF‐κB oncogenic events.

### 3.8 CD13/HDAC5 complex activates NF‐kB signaling via LSD1

**FIGURE 7:**
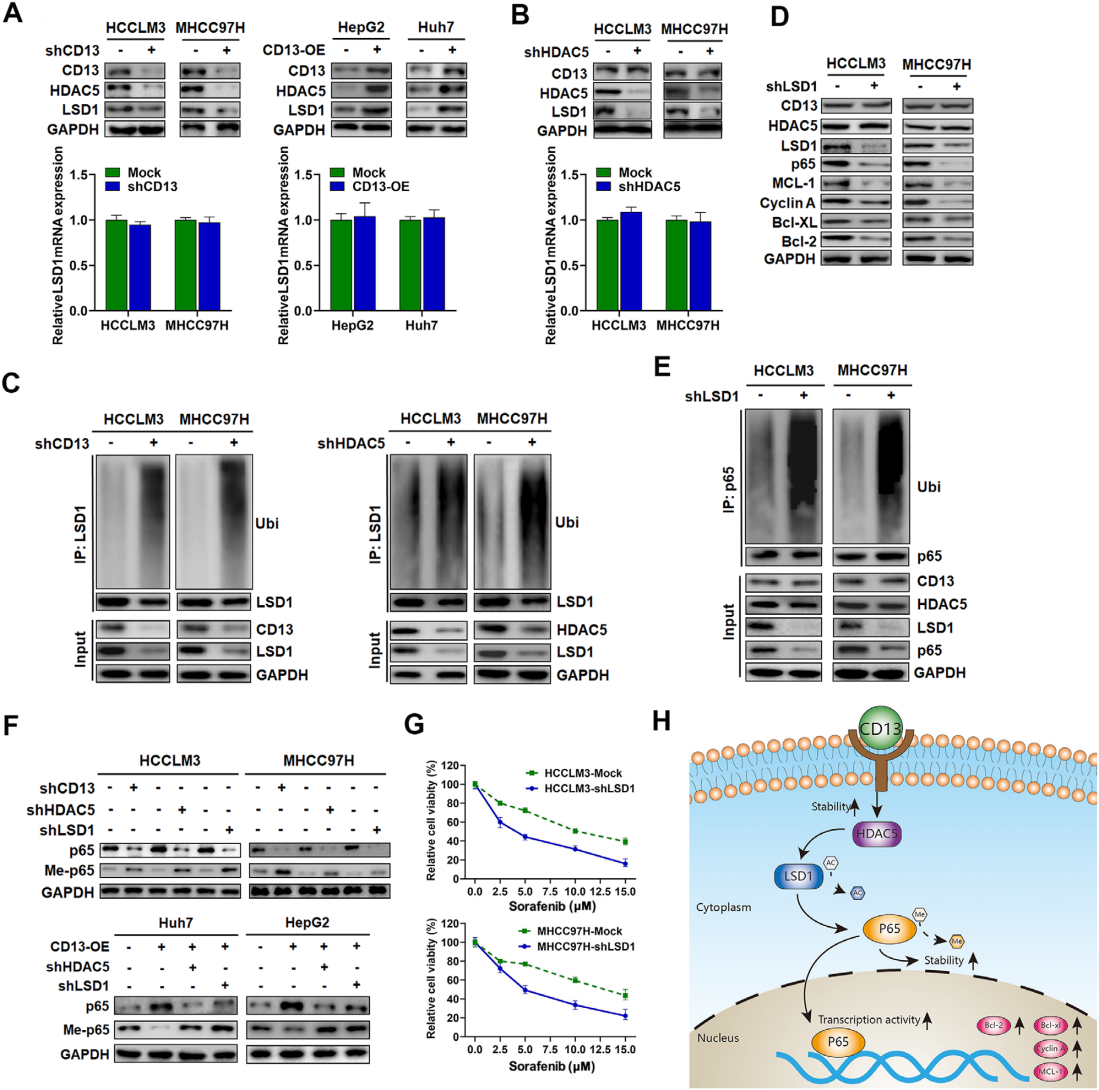
HDAC5 prevented p65 from degradation via deacetylating LSD1 in CD13‐high HCC cells. A, Effects of CD13 knockdown (left) and overexpression (right) on the protein expressions of LSD1 in indicated HCC cell. mRNA expressions showed no significant alteration due to CD13 expression modulations. B, Effects of HDAC5 knockdown on the protein expressions of LSD1 in indicated CD13‐high HCC cell. mRNA expressions showed no significant alteration due to HDAC5 expression modulations. C, Ubiquitination assay of LSD1 in HCC cells received indicated treatments. D, Effects of LSD1 knockdown on anti‐apoptotic and cell cycle gene expression states in CD13‐high HCC cells were assessed by WB assays. E, Ubiquitination assay of p65 in HCCLM3 (left) and MHCC97H (right) cells. F, WB assays showed methylation status of p65 were greatly enhanced due to CD13, HDAC5, or LSD1 knockdown, whereas demethylation effects of CD13 overexpression were abolished due to HDAC5 or LSD1 knockdown according to western blot. G, Effects of LSD1 knockdown on sorafenib susceptibility in CD13‐high HCC cells were assessed by CCK‐8 assays. H, A schematic diagram demonstrating molecular mechanisms underlying CD13 in HCC

Previous studies reported that HDAC5 could enhance the stability of LSD1, which was identified as a critical modifier for the demethylation of p65.^22^, ^23^ Therefore, we further explored whether CD13/HDAC5 complex could increase p65 protein level via stabilizing LSD1. As excepted, the inhibition of CD13 reduced LSD1 protein levels in HCC cell lines, whereas CD13 overexpression led to increased LSD1 levels (Figure 7A). Similarly, HDAC5 knockdown could induce decreased expression of LSD1 protein in HCCLM3 and MHCC97H (Figure 7B). However, there had no alteration of LSD1 mRNA with either CD13 knockdown or CD13 overexpression (Figure 7A,B). In addition, CD13 or HDAC5 depletion led to increased LSD1 ubiquitination, suggesting that CD13 and HDAC5 regulate LSD1 protein stability (Figure 7C).

The critical role of LSD1 in CD13/HDAC5‐mediating p65 expression was further explored, we found the expressions of p65 as well as NF‐kB‐associated genes were dramatically decreased after LSD1 depletion in HCCLM3 and MHCC97H (Figure 7D). Moreover, LSD1 knockdown could increase the level of p65 ubiquitination in HCCLM3 and MHCC97H (Figure 7E), supporting the vital role of LSD1 in maintaining p65 protein stability. Further results showed that LSD1 knockdown could dramatically increase methylated‐p65 level, accompanying with decreased total p65 protein expression (Figure 7F). These results indicate that LSD1 may serve as new demethylase for p65, which will need to be further investigated. Similar phenomenon could be observed after CD13 or HDAC5 was silenced. More importantly, silencing of LSD1 could abolish the demethylation effects of CD13 overexpression on Huh7 and HepG2 cells, which lead to a reduction of p65 protein (Figure 7F). Finally, cell proliferation by CCK‐8 assays showed that silencing of LSD1 sensitized HCCLM3 and MHCC97H cells to sorafenib treatment (Figure 7G). Together, our results demonstrated CD13/HDAC5 complex could upregulate p65 protein expression via stabilizing LSD1 in HCC.

## 4 DISCUSSION

In this study, we propose a model that CD13 is highly expressed in HCC, which leads to increased HDAC5 protein stability. HDAC5 promotes LSD1 deacetylation, which prevents it from protein ubiquitination and degradation. LSD1 enhances p65 protein demethylation and contributes to enhanced p65 protein stability and NF‐κB activation. Consequently, NF‐κB activation leads to gene activation involved in cell cycle and anti‐apoptosis, including but not limited to Cyclin A, BCL2, MCL‐1, and BCL‐xL (Figure 7H). Our study places the central role of CD13‐HDAC5‐LSD1‐NF‐κB signaling axis on contributing to the HCC tumorigenesis and sorafenib resistance.

CD13 has been proposed as a molecular marker of CSCs which sustains the long‐term clonal maintenance of the neoplasm. Recent studies have shown that compared with non‐CSCs, CSCs are more likely to survive in response to chemotherapy, indicating that targeting CSCs are clinically relevant.^7^, ^8^, ^24^ However, the existing studies have been focusing on the identification of surface markers to isolate this subgroup of CSCs and very little is known about the signaling pathway that regulates chemotherapy resistance. Our study uncovers high level of CD13, promotes HCC cell proliferation and invasion by forming a complex with HDAC5 protein, and prevents NF‐κB p65 subunit from being acetylated. Clinically, act as an independent predictor of recurrence and survival, CD13 is also involved in the development of sorafenib treatment resistance by regulating the expression of genes critical for cell proliferation, thereby evading cell cycle arrest. More importantly, targeting CD13 strategy showed great potential in reversing this resistance. Therefore, CD13 is of critical importance in the treatment of HCC.

Dysregulation of stem‐like signaling pathways maintains the CSCs in stem‐like properties, therefore accounting for enhanced tumorigenicity and therapy resistance in cancer. Inhibition of key signaling pathways that are used preferentially or uniquely by CSC constitutes a new therapeutic strategy for HCC,^11^ and this strategy showed promising prospective.^24^ Many works have been done to the correlation of CD13 and molecular and phenotypes characteristics, but little is known about the post‐transcriptional or translational regulation and molecular function of CD13.^25^, ^26^, ^27^, ^28^ In this study, we provide data to show that CD13 positively regulated proliferation, invasion, and chemoresistance of HCC cells in vitro and tumorigenic function in vivo. By our established HCC PDX platform, we found that HCC tumors with higher CD13 expression are more prone to grow tumor in the mice, suggesting CD13 plays a major role in driving tumor origin and growth. In present study, we found that overexpression of the CD13 protein was significantly correlated with elevated AFP, tumor encapsulation, microvascular invasion, and high CNLC stage. Interestingly, recent studies also indicated that CD13 positivity was an independent poor prognostic indicator for survival and recurrence in other cancers.^29^, ^30^, ^31^, ^32^ Consistently, our study demonstrated that CD13 is an important prognostic marker of poor outcome in patients, especially for patients with difficult predicted conventional clinical indexes, such as normal AFP and early‐stage disease.

Using the combination of IP with mass spectrometry (MS), we further confirmed CD13 forms a complex with HDAC5. By stabilizing NF‐κB p65 via HDAC5 deacetylase activity, CD13 activates the NF‐κB signaling targets. Downregulation of either CD13 or HDAC5 increased NF‐κB p65 acetylation (Lys310) and degradation, which lead to the decrease tumor growth. Notably, our data underlined that HDAC5‐LSD1‐p65 axis was mainly existed in CD13^+^ fractions, which suggested this post‐translational modification regulatory mechanism was restricted to CD13^+^ liver CSC. Based on the mechanism, we hypothesize the synergistic beneficial effects of ubenimex and the pan HDAC inhibitor Quisinostat, which was reported to inhibit cancer cells self‐renewal and the growth of medulloblastoma.^33^, ^34^ Tumor cells and xenografts exposed to the combination therapy show inhibited cellular proliferation characterized by lower levels of Cyclin A and D1. It is important to note that the combination therapy is also synergistic in the HCC metastatic PDX models, indicating that the novel combination treatment is not only effective in primary tumor but also metastatic foci. Our results identify a striking vulnerability to targeting approaches in HCC and will hopefully facilitate the rapid clinical evaluation of this new strategy.

Recently, pathway‐independent resistance mechanisms by CSCs contributing to therapeutic resistance have been identified.^35^, ^36^, ^37^ By using sorafenib‐sensitive/acquired resistant cell lines and PDX models, we first identify CD13 as a promoter for sorafenib resistance by upregulating cyclins (cyclin A and cyclin D1) and pro‐survival factors (Bcl‐2, Bcl‐XL and Mcl‐1) that is consistent with previous reports; and more importantly, CD13 inhibitor can overcome this resistance by re‐sensitizing tumors to sorafenib.^19^ Clinical data also indicated that high expression of CD13 correlates with a worse prognosis in HCC patients following sorafenib treatment. All of these implied that CD13 might be a useful biomarker for predicting the response to sorafenib therapy for HCC patients. Several studies have confirmed that targeting CD133 or CD47 in combination with sorafenib may represent a novel and effective treatment in HCC,^38^, ^39^, ^40^, ^41^ while there are no available drugs in a clinical setting. However, ubenimex (Nippon Kayaku Co, Tokyo) is approved in Japan as an antitumor drug, which may be easy to facilitate clinical translation of the combination therapy.

Our study has some limitations. The study is a retrospective study from a single medical center, the prognostic significance of CD13 still needs to be validated by a prospective, multi‐center, clinical study. The expression of CD13 on tumor endothelial cells and its clinical significance have been reported in other previous studies.^30^, ^42^ However, we cannot differentiate tumor cells and tumor endothelial cells based on IHC analysis in this study. Meanwhile, we confirmed that CD13 was mainly expressed in tumor cells and acted as a powerful indicator for predicting poor prognosis in HCC. Thus, the biological function of CD13 in tumor endothelial cells was not focused in this research, and we may do it in our further investigation.

## 5 CONCLUSIONS

Overall, our study identified that CD13 as a regulator in cancer progression and sorafenib resistance by forming CD13/HDAC5 complex and regulating NF‐κB p65 acetylation. Given that the synergistic effect of CD13 and HDAC5 inhibitors, we hope that our findings will motivate more clinical trials in HCC that involve these inhibitors. More importantly, CD13 inhibitor could reverse sorafenib resistance and enhance the effect of sorafenib. High expression of CD13 indicated shorter OS and more rapid recurrence for HCC patients undergoing surgical resection. Thus, further exploration of CD13‐mediated signaling pathways would allow us to elucidate the mechanisms of CSCs involving in HCC tumorigenesis and chemoresistance, therefore guiding better treatments for this lethal cancer.

## ETHICS APPROVAL AND CONSENT TO PARTICIPATE

Present study was performed in accordance with the 1975 Declaration of Helsinki. Approval for the use of human subjects was obtained from the Research Ethical Committee of Zhongshan Hospital, and informed consent was obtained from each individual enrolled in this study.

## COMPETING INTERESTS

The authors declare that there is no conflict of interest that could be perceived as prejudicing the impartiality of the research reported.

## AUTHOR CONTRIBUTIONS

Study concept and design: Hu, Xu, Li, and Yang. Analysis and interpretation of data: Hu, Xu, Huang, Cheng, Guo, Yin, and Gao. Drafting of the manuscript: Hu, Xu, Yuan‐Cheng Li, Wang, and Wu. Critical revision of the manuscript for important intellectual content: Yang, Zhou, and Fan. Approval of the final manuscript: Hu, Xu, Li, Huang, Cheng, Guo, Yin, Gao, Wang, Wu, Zhou, Fan, and Yang.

## Supporting information

Supporting Information

Supporting Information

Supporting Information

Supporting Information

Supporting Information

Supporting Information

Supporting Information

Supporting Information

Supporting Information

Supporting Information

Supporting Information

Supporting Information

## Data Availability

All data generated or analyzed during this study are included in this published article (and also in Supporting Information).
