# Supplementary material for: CD13 promotes hepatocellular carcinogenesis and sorafenib resistance by activating HDAC5‐LSD1‐NF‐κB oncogenic signaling
Source: Clin Transl Med. 2020 Dec 1;10(8):e233. doi: 10.1002/ctm2.233 (PMC7708822; doi:10.1002/ctm2.233)
Supplement: Supplementary file 1 — Supporting Information [file CTM2-10-e233-s001.docx]

**CD13 promotes hepatocellular carcinogenesis and sorafenib resistance by activating HDAC5-LSD1-NF-κB oncogenic signaling**

## Supplementary Figures

**Supplementary Figure 1.** (**A**) qRT-PCR and western blots of CD13 expression in HCCLM3 and MHCC97H cells stably infected with the virus encoding either mock or CD13 shRNA or HepG2 and Huh7 cells infected with the virus encoding either mock or CD13 expression. (**B**) CD13 knock down significantly restrained MHCC97H proliferation capacities, whereas CD13 overexpression greatly promoted HepG2 proliferation in HCC according to CCK-8 assays. (**C**) CD13 knock down resulted in G0/G1 arrest in MHCC97H cell according to cell cycle determinations. (**D**) CD13 knock down significantly inhibited MHCC97H invasiveness capacities, whereas CD13 overexpression greatly enhanced HepG2 invasion in HCC according to Transwell assays.

**Supplementary Figure 2.** Representative images of tumors in nude mice bearing HCCLM3, HCCLM3 shCD13, Huh7 and Huh7 CD13; n = 6.

**Supplementary Figure 3.** The Kaplan-Meier analysis of overall survival (OS) and time to recurrence (TTR) for the CD13 in AFP 20 ng/mL (A) and CNLC I (B) groups.

**Supplementary Figure 4.** (**A**) qRT-PCR for CD13 in four HCC cell lines (MHCC97H, MHCC97H-SR, Huh7 and Huh7-SR). (**B**) Macrograph of tumors in all groups. (**C**) qRT-PCR for CD13, BCL-2, BCL-XL and MCL-1 in HCC cell lines treated with control and ubenimex (0.25 mg/ml). (**D**) qRT-PCR for cyclin A, D1, B1 and CDC25 in HCC cell lines treated with control and ubenimex (0.25 mg/ml). **P* < 0.05, ****P* < 0.001.

**Supplementary Figure 5.** (**A**) Protein and mRNA expressions of p65 and p-p65 were detected by WB and RT-PCR assays, respectively. (**B**) p65 knockdown restored sorafenib susceptibility in HCCLM3 and MHCC97H cells according to CCK-8 assays. (**C**) Effects of p65 knockdown on anti-apoptotic and cell cycle gene expression states in HCC cells were assessed by WB assays.

**Supplementary Figure 6.** Interaction was detected between CD13 and p65 according to Co-IP.

## Supplementary Tables

**Supplementary Table 1.** Sequence of primers for qRT-PCR

**Supplementary Table 2.** Information of primary antibodies.

**Supplementary Table 3.** The clinicopathological characteristics of patients in the training cohorts**.**

**Supplemenrary Table 4.** The clinico-pathologic characteristics of patients**.**

**Supplemenrary Table 5.** A total of 180 proteins were identified by LC-MS.

## Supplementary Materials and Methods

### Immunoprecipitation (IP), LC-MS/MS Mass Spectrometry and Co-immunoprecipitation (Co-IP)

For IP assay, Myc-CD13 was transiently transfected into HEK293 cells, and at 48 hours post transfection, the cells were lysed in buffer A (50 mM Tris, 150 mM NaCl, 5 mM EDTA, 0.5% NP-40 and 0.1% Triton X-100) supplemented with 1 mM NaF, 1 mM Na_2_VO_3_, 1 mM phenylmethanesulfonylfluoride, 1 μg/ml aprotonin, 1 μg/ml leupeptin and 1 μg/ml pepstatin. IP was performed with anti-Myc antibody with Pierce™ Classic Magnetic IP/Co-IP Kit (#88804, Thermo, USA) according to manufacturer’s instructions. After collecting precipitates, WB assays were conducted with indicated antibodies to verify the efficiencies of IP assay. Subsequently, LC-MS/MS mass spectrometry was performed to identify critical interactor of CD13 in HCC cells as previous studies did. The cells were separated into fractions, and the supernatants derived from each fraction were detected through silver staining. HDAC5 was identified through LC-MS/MS mass spectrometry. The Western blotting and IP analyses were previously described [1].

Co-IP analyses were performed as described previously [2]. Briefly, HCCLM3 cells were transfected with indicated expression plasmids. Cell lysates were prepared 48 hours after transfection in lysis buffer containing 20 mM Tris-HCl (pH 8.0),150 mM NaCl, 0.5% NP-40, 2.5 mM EDTA, and protease inhibitor mixture. The whole-cell extracts were precleared with protein A/G beads, and co-IP assays were performed with either Myc or Flag antibodies.

### Matrigel Invasion assay, CCK-8 assay, Colony Formation assay, and Cell Cycle analysis

Matrigel invasion and cell proliferation assays were performed as previously described [3]. Briefly, 2×10 ^6^ HCC cells from each group were seeded in the upper chamber, which was coated with a matrix gel (1:8 diluted, Corning, ME) with FBS-free DMEM. DMEM containing 10% FBS was added to the lower chamber as chemo-attraction. Mitomycin c was added in the upper chamber to stop cell proliferation. After 24 h of incubation, the cells that had invaded to the lower surface of membrane were fixed with 4% methanol, stained with crystal violet, and counted in 10 random × 100 microscopic fields per sample.

CCK-8 and colony formation assays were conducted to evaluate the influence of CD13 on HCC proliferation. CCK-8 assays were performed as the previous study [4]. Briefly, tumor cells were seeded in 96-well plate at a density of 1000 cells per well with DMEM supplemented with 10% FBS. The absorbance of 450 nm was detected every 24 hours to generate growth curves. Colony formation assay was performed as previously described. Briefly, tumor cells were seeded in a 6-well plate at a density of 1000 per well and cultured at 37 °C for two weeks. Afterwards, cells were fixed with 4% paraformaldehyde and stained with 0.1% crystal violet. The colony-forming ability was assessed by counting the number of colonies (>70 cells) under a microscope after staining with crystal violet (#C0775, Sigma-Aldrich, USA). Representative images were photographed using an Olympus LX-71 fluorescence microscope.

Cell cycle assessments were conducted via FACS approach. Briefly, indicated HCC cells were resuspended in 10 ml PBS and fixed in 100% ethanol overnight at 4 °C. The fixed cells were pelleted by centrifugation, resuspended in 1 ml PBS containing ribonuclease A (100 mg/ml) and incubated for 30 min at 37 °C. Then, propidium iodide (4 mg/ml) was added to the samples followed by FACS analysis on a FACSCalibur (BD Bioscience, San Jose, CA, USA). All above experiments were performed in triplicate.

### Construction of Tissue Microarrays and immunohistochemistry (IHC).

Tumor sections were processed with an antigen retrieval step before they were incubated with antibodies overnight at 4°C. These slides were then washed three times with PBS, incubated with horseradish peroxidase-labeled anti-rabbit antibody for 30 min at 37°C, and developed with diaminobenzidine tetrahydrochloride and hydrogen peroxide in PBS.

The immunoreactive score system (IRS) has been described previously [5]. Briefly, staining extent score was on a scale of 0-4, corresponding to the percentage of immunoreactive tumor cells (0%, 1%-5%, 6%-25%, 26%-75%, and 76%-100%, respectively). Staining intensity was scored as negative (score = 0), weak (score = 1), or strong (score = 2). A score ranging from 0-8 was calculated by multiplying the staining extent score with the intensity score, resulting in a low (0-4) level or a high (＞4) level value for each specimen.

### Cell line derived xenografts and PDX models

HCCLM3, HCCLM3-shCD13, Huh7 or Huh7-shCD13 cells (1 X 10^6^) in 0.2 mL serum-free culture medium were injected subcutaneously into three 4- to 6-week old male athymic BALB/c nu/nu mice (Shanghai Institute of Material Medical, Chinese Academy of Science). The mice were housed at the Zhongshan Hospital of Fudan University. After 4 weeks, the mice were sacrificed by overdosage of pentobarbital and the lung sections were stained with hematoxylin and eosin (H&E). Tumor growth was recorded three times per week by measuring the tumor length (L) and width (W) with a caliper. Tumor volume in cm^3^ was calculated as 0.5×L×W^2^.

PDX models were established as previously reported [5]. When the tumor implanted into the livers of 24 mice, the mice were randomly divided into four groups according to treatment: 1) control (PBS, 100 ml, intraperitoneal injection), 2) ubenimex (4 mg/kg, daily oral administration), 3) sorafenib (30 mg/kg, daily oral administration), and 4) ubenimex plus sorafenib (administered as described for single agent treatments).

## References

1. Yang L-X, Gao Q, Shi J-Y, et al. Mitogen-activated protein kinase kinase kinase 4 deficiency in intrahepatic cholangiocarcinoma leads to invasive growth and epithelial-mesenchymal transition. Hepatology (Baltimore, Md). 2015; 62: 1804-16.

2. Jia H, Cong Q, Chua JFL, et al. p57Kip2 is an unrecognized DNA damage response effector molecule that functions in tumor suppression and chemoresistance. Oncogene. 2015; 34: 3568-81.

3. Hu B, Sun D, Sun C, et al. A polymeric nanoparticle formulation of curcumin in combination with sorafenib synergistically inhibits tumor growth and metastasis in an orthotopic model of human hepatocellular carcinoma. Biochem Biophys Res Commun. 2015; 468: 525-32.

4. Ma X-L, Shen M-N, Hu B, et al. CD73 promotes hepatocellular carcinoma progression and metastasis via activating PI3K/AKT signaling by inducing Rap1-mediated membrane localization of P110β and predicts poor prognosis. J Hematol Oncol. 2019; 12: 37.

5. Jiang Y, Sun A-H, Zhao Y, et al. Proteomics identifies new therapeutic targets of early-stage hepatocellular carcinoma. Nature. 2019; 567: 257-61.
