# Supplementary figures and images for: CD13 promotes hepatocellular carcinogenesis and sorafenib resistance by activating HDAC5‐LSD1‐NF‐κB oncogenic signaling
Source: Clin Transl Med. 2020 Dec 1;10(8):e233. doi: 10.1002/ctm2.233 (PMC7708822; doi:10.1002/ctm2.233)

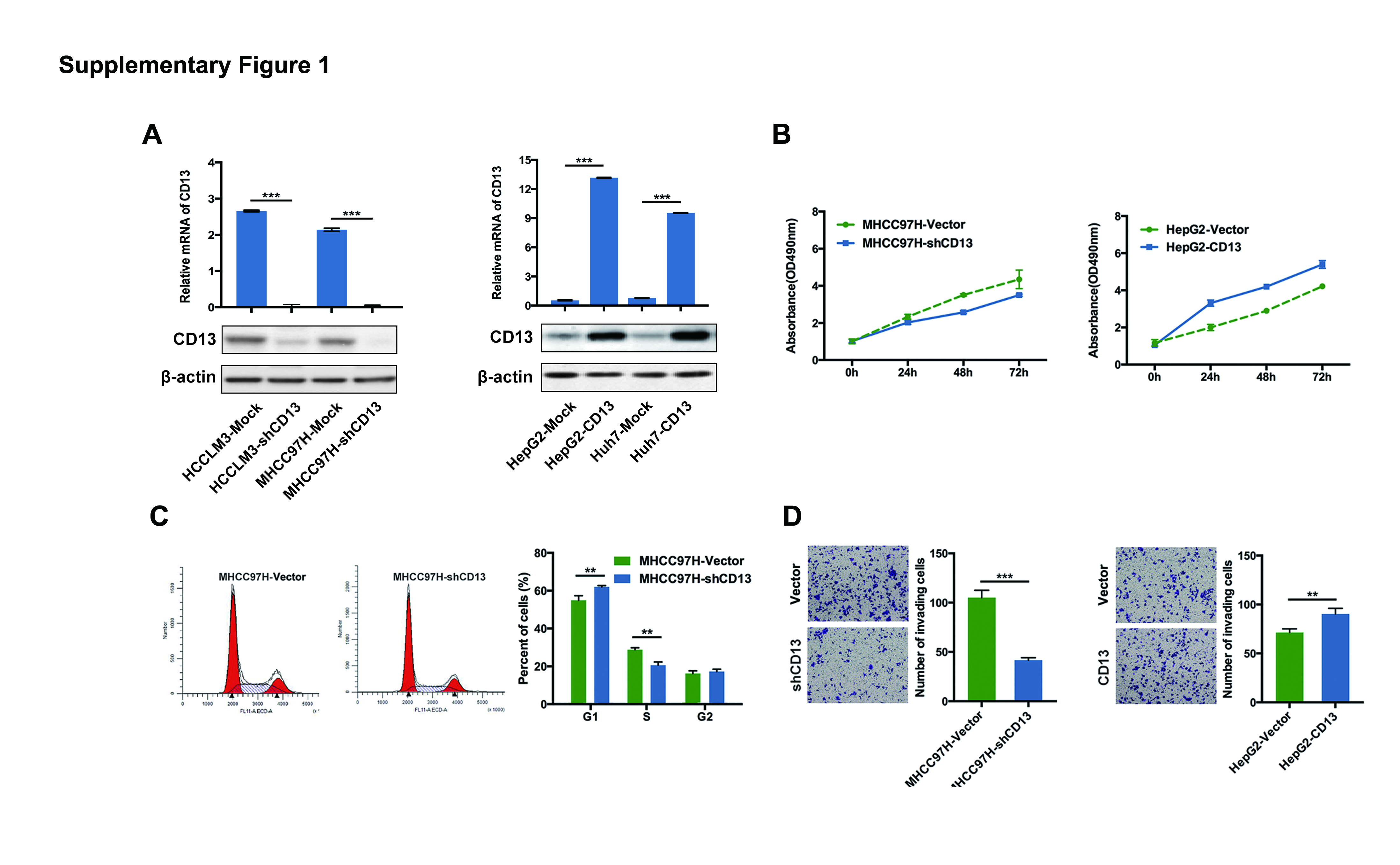

Supplement: Supplementary file 2 — Supporting Information [file CTM2-10-e233-s002.tif]

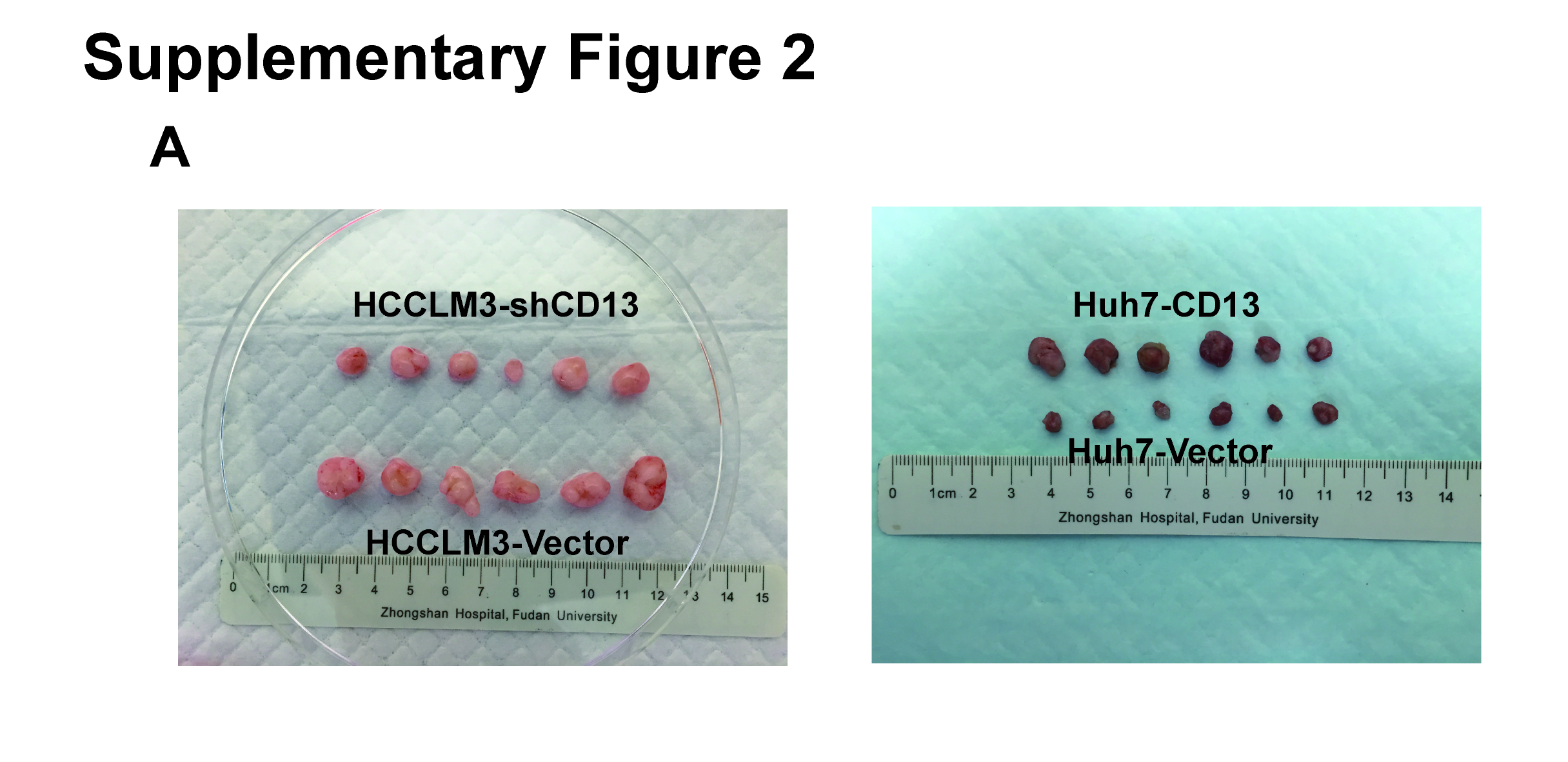

Supplement: Supplementary file 3 — Supporting Information [file CTM2-10-e233-s003.tif]

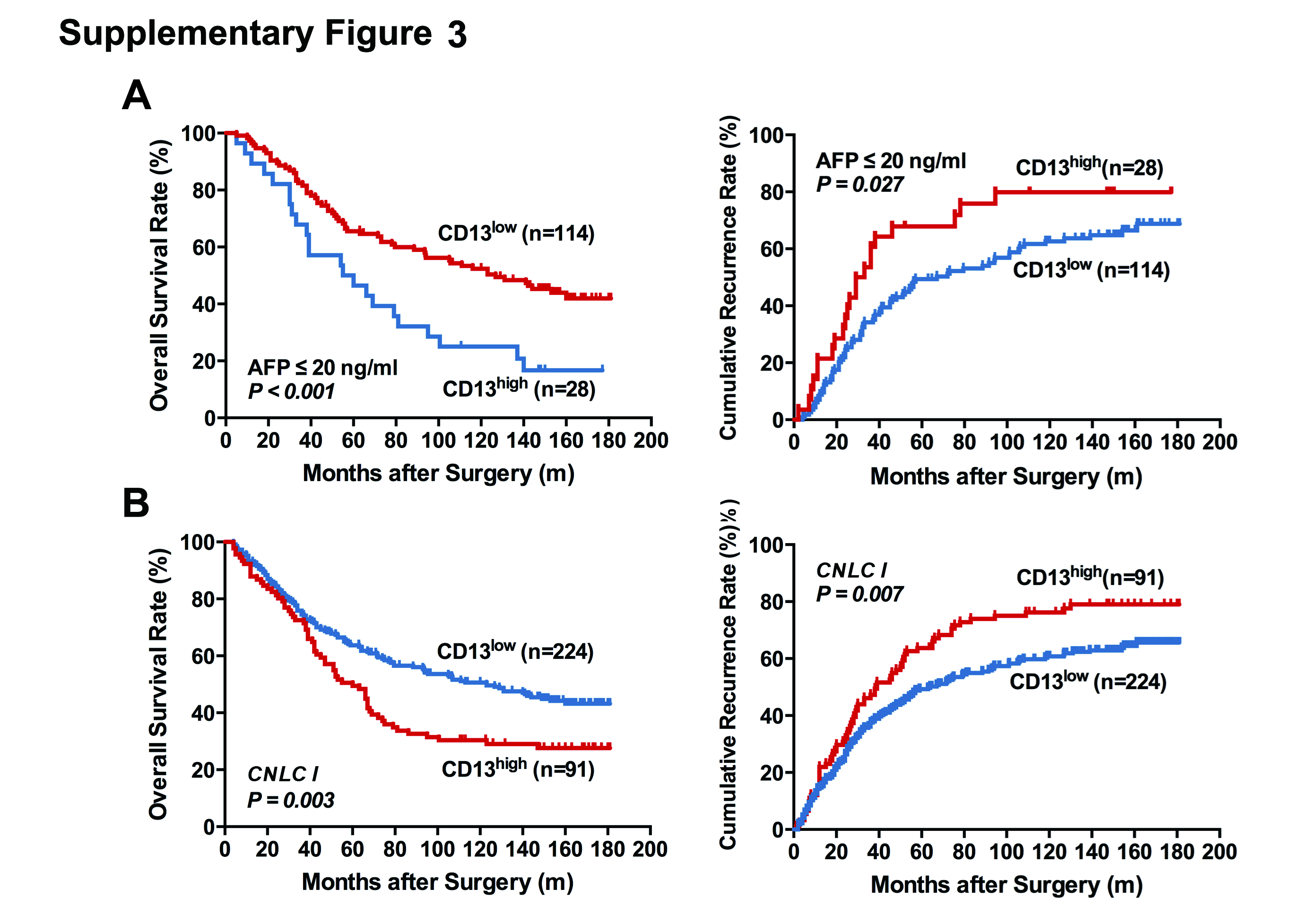

Supplement: Supplementary file 4 — Supporting Information [file CTM2-10-e233-s004.tif]

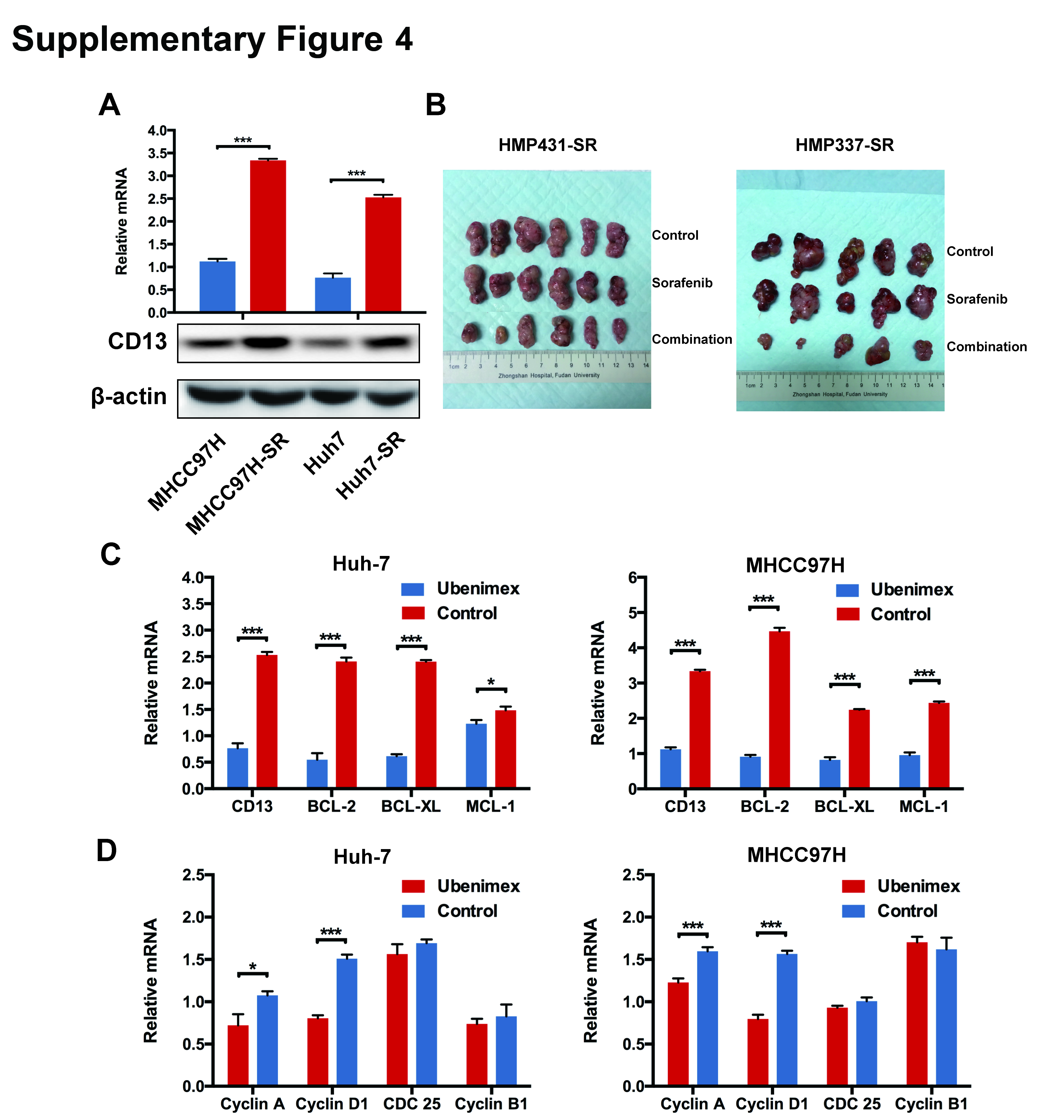

Supplement: Supplementary file 5 — Supporting Information [file CTM2-10-e233-s005.tif]

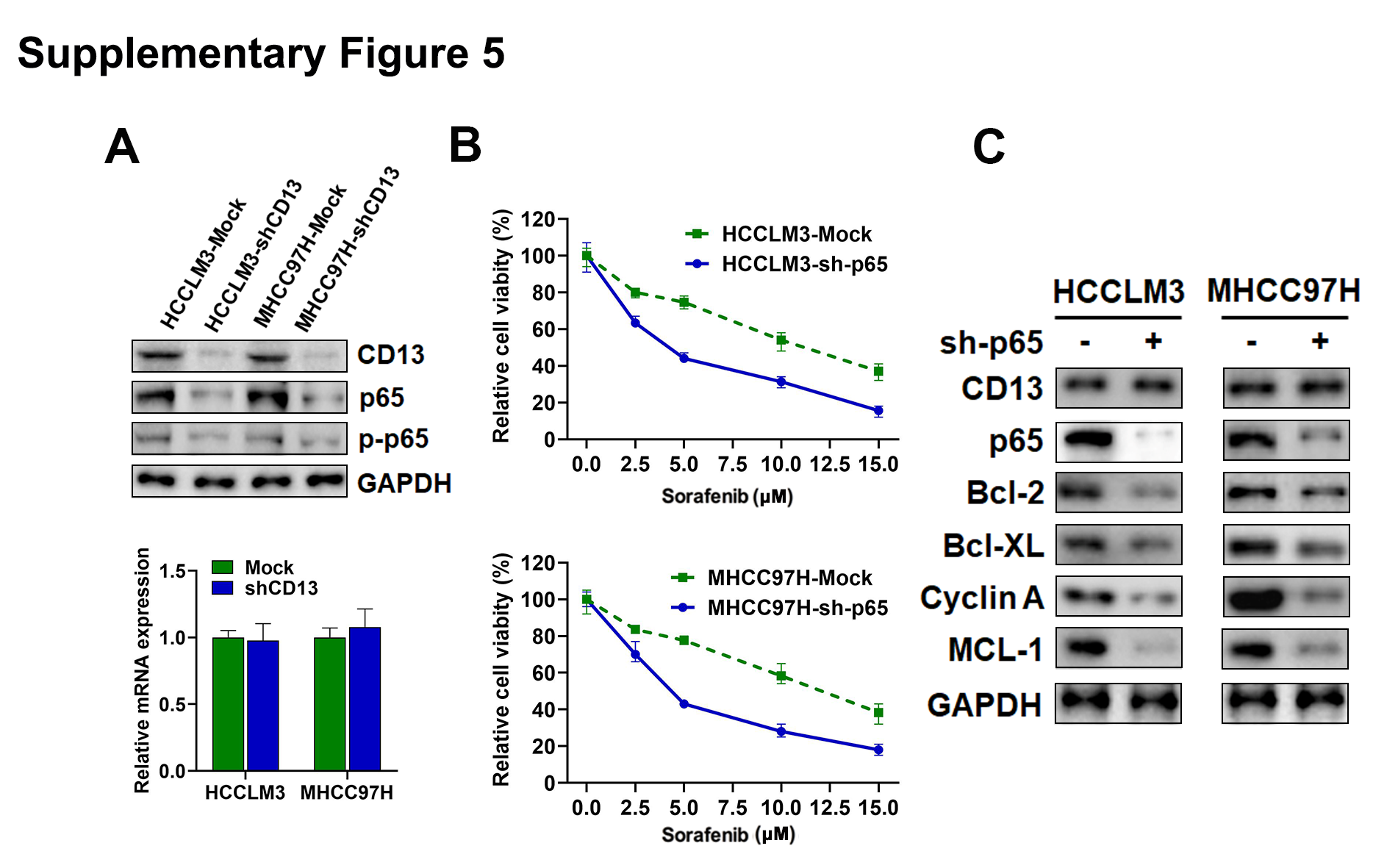

Supplement: Supplementary file 6 — Supporting Information [file CTM2-10-e233-s006.tif]

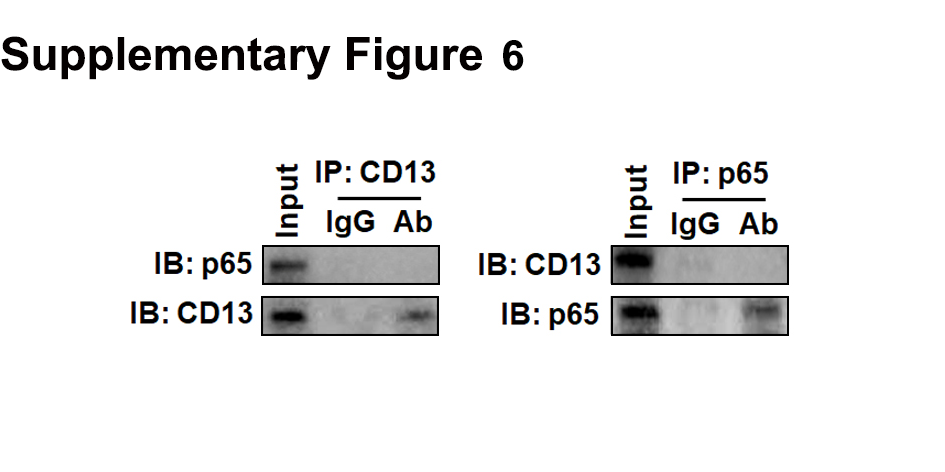

Supplement: Supplementary file 7 — Supporting Information [file CTM2-10-e233-s007.tif]
