# Supplementary material for: CD13 promotes hepatocellular carcinogenesis and sorafenib resistance by activating HDAC5‐LSD1‐NF‐κB oncogenic signaling
Source: Clin Transl Med. 2020 Dec 1;10(8):e233. doi: 10.1002/ctm2.233 (PMC7708822; doi:10.1002/ctm2.233)
Supplement: Supplementary file 8 — Supporting Information [file CTM2-10-e233-s008.docx]

| **Supplementary Table 1. Sequence of primers for qRT-PCR** | | |
| --- | --- | --- |
| **Gene** | **Forward primer (5'…3')** | **Reverse primer (5'…3')** |
| CD13 | TTCAACATCACGCTTATCCACC | AGTCGAACTCACTGACAATGAAG |
| HDAC5 | TCCTGTTCGCTGAGTTCCAGAA | CCTCAGCTTTACCTCAGTGCTG |
| CDC25 | CCAACAACACCCAAATCAGTC | TGCGGTTATTGTCCAGTTCA |
| CyclinA | AGTGGAGTTGTGCTGGCTAC | AGTCAGGGAGTGCTTTCTTT |
| CyclinB1 | AAATAAGGCGAAGATCAACA | AGAGGCAGTATCAACCAAAA |
| CyclinD1 | GCTGCGAAGTGGAAACCATCC | CATTTGAAGTAGGACACCGAGGG |
