# Supplementary material for: CD13 promotes hepatocellular carcinogenesis and sorafenib resistance by activating HDAC5‐LSD1‐NF‐κB oncogenic signaling
Source: Clin Transl Med. 2020 Dec 1;10(8):e233. doi: 10.1002/ctm2.233 (PMC7708822; doi:10.1002/ctm2.233)
Supplement: Supplementary file 9 — Supporting Information [file CTM2-10-e233-s009.docx]

| **Supplementary Table 2. Information of primary antibodies** | | | |
| --- | --- | --- | --- |
| **Target protein** | **Manufacturer** | **Application** | **Dilution** |
| CD13 | Abcam (ab108310) | WB | 1:1000 |
|  |  | IHC | 1:200 |
|  | Abcam (ab108382) | IP | 1:20 |
| Bcl-2 | Abcam (ab182858) | WB | 1:1000 |
| Bcl-XL | Abcam (ab178844) | WB | 1:1000 |
| Mcl-1 | Abcam (ab186822) | WB | 1:1000 |
| Cycilin A1 | Abcam (ab53699) | WB | 1:1000 |
|  |  | IHC | 1:150 |
| Cyclin D1 | Abcam (ab40754) | WB | 1:800 |
|  |  | IHC | 1:100 |
| CDC25 | Abcam (ab247941) | WB | 1:800 |
| Cyclin B1 | Abcam (ab32053) | WB | 1:1000 |
| p65 | CST (#8242) | WB | 1:1000 |
|  |  | IP | 1:20 |
| p-p65 | CST (#3033) | WB | 1:1000 |
| HA-E2F1 | CST (#3742) | WB | 1:1000 |
| Flag | CST (#14793) | WB | 1:1000 |
| Myc | CST (#2276) | IP | 1:20 |
| HDAC5 | CST (#20458) | WB | 1:1000 |
|  |  | IP | 1:1000 |
| Ubiquitin | Abcam (ab7780) | WB | 1:1000 |
| LSD1 | Abcam (ab17721) | WB | 1:20 |
|  |  | IP | 1:1000 |
| Me-p65 | Lifespan(LS-C380562) | WB | 1:1000 |
| β-Actin | CST (#4970) | WB | 1:1000 |
