# Supplementary material for: CD13 promotes hepatocellular carcinogenesis and sorafenib resistance by activating HDAC5‐LSD1‐NF‐κB oncogenic signaling
Source: Clin Transl Med. 2020 Dec 1;10(8):e233. doi: 10.1002/ctm2.233 (PMC7708822; doi:10.1002/ctm2.233)
Supplement: Supplementary file 10 — Supporting Information [file CTM2-10-e233-s010.docx]

| **Supplementary Table 3. The clinicopathological characteristics of patients in the training cohorts** | | | | | | | |
| --- | --- | --- | --- | --- | --- | --- | --- |
| Clinical and pathological Indexes | |  | CD13^low^ (n=277) |  | CD13^high^ (n=126) |  | *P* |
|  |  |  |  |  |  |  |  |
| Age (y) | ≤50 |  | 138 |  | 65 |  | 0.742 |
|  | >50 |  | 139 |  | 61 |  |  |
| Sex | Female |  | 34 |  | 16 |  | 0.905 |
|  | Male |  | 243 |  | 110 |  |  |
| Liver cirrhosis | No |  | 52 |  | 22 |  | 0.752 |
|  | Yes |  | 225 |  | 104 |  |  |
| Child-Pugh | A |  | 264 |  | 120 |  | 0.976 |
|  | B |  | 13 |  | 6 |  |  |
| HBsAg | Negative |  | 55 |  | 18 |  | 0.178 |
|  | Positive |  | 222 |  | 108 |  |  |
| HCV | Negative |  | 270 |  | 123 |  | 0.930 |
|  | Positive |  | 7 |  | 3 |  |  |
| GGT (U/l) | ≤54 |  | 120 |  | 44 |  | 0.112 |
|  | >54 |  | 157 |  | 82 |  |  |
| ALT (U/l) | ≤75 |  | 248 |  | 116 |  | 0.425 |
|  | >75 |  | 29 |  | 10 |  |  |
| AFP (ng/ml) | ≤20 |  | 114 |  | 28 |  | **<0.001** |
|  | >20 |  | 163 |  | 98 |  |  |
| Tumor encapsulation | Complete |  | 171 |  | 64 |  | **0.039** |
|  | None |  | 106 |  | 62 |  |  |
| Tumor differentiation | I-II |  | 177 |  | 86 |  | 0.395 |
|  | III-IV |  | 100 |  | 40 |  |  |
| Tumor size (cm) | ≤5 |  | 163 |  | 70 |  | 0.535 |
|  | >5 |  | 114 |  | 56 |  |  |
| Tumor number | Single |  | 225 |  | 98 |  | 0.421 |
|  | Multiple |  | 52 |  | 28 |  |  |
| Vascular invasion | No |  | 218 |  | 82 |  | **0.004** |
|  | Yes |  | 59 |  | 44 |  |  |
| CNLC | I |  | 224 |  | 91 |  | 0.052 |
|  | II-III |  | 53 |  | 35 |  |  |
| Abbreviations: HBsAg, hepatitis B surface antigen; HCV, hepatitis C virus; GGT, gamma-glutamyl transferase; ALT, alanine aminotransferase; AFP, alpha-fetoprotein; CNLC, China liver cancer staging. | | | | | | | |
