# Supplementary material for: CD13 promotes hepatocellular carcinogenesis and sorafenib resistance by activating HDAC5‐LSD1‐NF‐κB oncogenic signaling
Source: Clin Transl Med. 2020 Dec 1;10(8):e233. doi: 10.1002/ctm2.233 (PMC7708822; doi:10.1002/ctm2.233)
Supplement: Supplementary file 11 — Supporting Information [file CTM2-10-e233-s011.docx]

| **Supplemenrary Table 4. The clinico-pathologic characteristics of patients** | | | | | | | | |
| --- | --- | --- | --- | --- | --- | --- | --- | --- |
| Clinical and pathological indexes | |  | Total |  | CD13 | |  | *P* value |
|  |  |  |  |  | Low | High |  |  |
| Age (y) | ≤50 |  | 24 |  | 10 | 14 |  | 0.175 |
|  | >50 |  | 21 |  | 13 | 8 |  |  |
| Sex | Female |  | 9 |  | 1 | 8 |  | 0.317^#^ |
|  | Male |  | 36 |  | 22 | 14 |  |  |
| Liver cirrhosis | No |  | 21 |  | 11 | 10 |  | 0.873 |
|  | Yes |  | 24 |  | 12 | 12 |  |  |
| HBsAg | Negative |  | 3 |  | 0 | 3 |  | 0.217^#^ |
|  | Positive |  | 42 |  | 23 | 19 |  |  |
| GGT (U/l) | ≤54 |  | 16 |  | 9 | 7 |  | 0.608 |
|  | >54 |  | 29 |  | 14 | 15 |  |  |
| ALT (U/l) | ≤40 |  | 21 |  | 11 | 10 |  | 0.873 |
|  | >40 |  | 24 |  | 12 | 12 |  |  |
| AFP (ng/ml) | ≤400 |  | 29 |  | 17 | 12 |  | 0.175 |
|  | >400 |  | 16 |  | 6 | 10 |  |  |
| Metastasis | No |  | 36 |  | 19 | 17 |  | 0.941^#^ |
|  | Yes |  | 9 |  | 4 | 5 |  |  |
| ^#^Continuity correction | | | | | | | | |
| Abbreviations: HBsAg, hepatitis B surface antigen; GGT, gamma-glutamyl transferase; ALT, alanine aminotransferase; AFP, alpha-fetoprotein | | | | | | | | |
